# In situ identification of the metallic state of Ag nanoclusters in oxidative dispersion

**DOI:** 10.1038/s41467-021-21552-2

**Published:** 2021-03-03

**Authors:** Rongtan Li, Xiaoyan Xu, Beien Zhu, Xiao-Yan Li, Yanxiao Ning, Rentao Mu, Pengfei Du, Mengwei Li, Huike Wang, Jiajie Liang, Yongsheng Chen, Yi Gao, Bing Yang, Qiang Fu, Xinhe Bao

**Affiliations:** 1 State Key Laboratory of Catalysis, Dalian Institute of Chemical Physics, The Chinese Academy of Sciences, Dalian, China; 2 University of Chinese Academy of Sciences, Beijing, China; 3 CAS Key Laboratory of Science and Technology on Applied Catalysis, Dalian Institute of Chemical Physics, The Chinese Academy of Sciences, Dalian, China; 4 Dalian National Laboratory for Clean Energy, Dalian Institute of Chemical Physics, The Chinese Academy of Sciences, Dalian, China; 5 Interdisciplinary Research Center, Shanghai Advanced Research Institute, The Chinese Academy of Sciences, Shanghai, China; 6 Key Laboratory of Interfacial Physics and Technology, Shanghai Institute of Applied Physics, The Chinese Academy of Sciences, Shanghai, China; 7 School of Materials Science and Engineering, National Institute for Advanced Materials, Nankai University, Tianjin, China; 8 Key Laboratory of Functional Polymer Materials of Ministry of Education, College of Chemistry, Nankai University, Tianjin, China

**Keywords:** Heterogeneous catalysis, Surface chemistry

## Abstract

Oxidative dispersion has been widely used in regeneration of sintered metal catalysts and fabrication of single atom catalysts, which is attributed to an oxidation-induced dispersion mechanism. However, the interplay of gas-metal-support interaction in the dispersion processes, especially the gas-metal interaction has not been well illustrated. Here, we show dynamic dispersion of silver nanostructures on silicon nitride surface under reducing/oxidizing conditions and during carbon monoxide oxidation reaction. Utilizing environmental scanning (transmission) electron microscopy and near-ambient pressure photoelectron spectroscopy/photoemission electron microscopy, we unravel a new adsorption-induced dispersion mechanism in such a typical oxidative dispersion process. The strong gas-metal interaction achieved by chemisorption of oxygen on nearly-metallic silver nanoclusters is the internal driving force for dispersion. In situ observations show that the dispersed nearly-metallic silver nanoclusters are oxidized upon cooling in oxygen atmosphere, which could mislead to the understanding of oxidation-induced dispersion. We further understand the oxidative dispersion mechanism from the view of dynamic equilibrium taking temperature and gas pressure into account, which should be applied to many other metals such as gold, copper, palladium, etc. and other reaction conditions.

## Abstract (web summary)

Designing ultra small metal nanoclusters or single atoms with metallic state is a challenge. Here, the authors demonstrate the stabilization of ultra small silver clusters in the nearly-metallic state by oxygen adsorption at high temperature, using in situ spectroscopy and microscope technologies.

## Introduction

Sintering is a longstanding problem in heterogeneous catalysis leading to deactivation after the long-term operation at high temperatures^1–3^. Many regeneration methods are developed to redisperse the sintered metal catalysts, such as oxidation–reduction, chlorination–oxychlorination, thermal treatment with halohydrocarbons, etc^2,4–6^. Among those, oxidative dispersion is one widely adopted process for the regeneration of used industrial catalysts^6–11^. It has been shown that sintered metal catalysts can break apart into highly dispersed oxide segments during oxidation and are then reactivated by reduction, yielding metal nanoclusters with restored activity^4^. Beyond catalyst regeneration, oxidative dispersion has recently been employed as one important route to fabricate single-atom catalysts (SACs) and oxidation treatment of supported metal catalysts in the oxidative atmosphere such as air, O_2_, and water vapor produces SACs of Pt, Pd, Rh, Ag, etc^12–18^. Hence, oxidative dispersion is of paramount importance for the fabrication and regeneration of highly/atomically dispersed catalysts with remarkable activity.

Dispersion under oxidizing conditions is a common phenomenon reported in catalysts of Rh/CeO_2_, Pt/CHA(siliceous chabazite), Ru/CeO_2_, Ag/MnO_*x*_, Pt/FeO_*x*_, etc^6,12,13,19,20^. The consensus on the oxidative dispersion process includes the formation of mobile metal oxide species from large metal particles and capture of these species on the support surface, which are regarded as the most crucial steps in the so-called oxidation-induced dispersion mechanism as revealed by in situ electron microscopy and/or in situ spectroscopy^21–28^. For example, Nagai et al. ascribed the redispersion of Pt nanoparticles to the formation of Pt oxide species as observed in O_2_ at 873 K by in situ X-ray absorption spectroscopy (XAS)^22^. Nevertheless, the underlying mechanism yet needs to be fully depicted. It is well known that the oxidation of metals is strongly dependent on both oxidant partial pressure and temperature. The high temperature applied in catalyst treatments, in general, does not favor the oxide formation, particularly for metals with small heat of formation of an oxide such as Au, Ag, Pd, etc^29–31^. Thus, more detailed investigation by in situ characterization is highly demanded in order to provide new and deep insights into the dynamic mechanism for the oxidative dispersion of supported metal catalysts.

In this work, we performed a state-of-the-art study for the dynamic dispersion of Ag aggregates i.e., nanowires (NWs) and nanoparticles (NPs) supported on Si_3_N_4_ surfaces upon heating in O_2_ using a combination of ex situ, quasi in situ, and in situ characterization techniques including near-ambient pressure XPS (NAP-XPS), near-ambient pressure photoemission electron microscopy (NAP-PEEM), and environmental scanning (transmission) electron microscopy (ESEM/ESTEM). The oxidative dispersion of Ag aggregates has been unambiguously observed, which occurs via the formation of mobile metallic Ag nanoclusters rather than Ag oxide species, and remarkably enhances their catalytic reactivity in CO oxidation. Chemisorption of oxygen from mbar O_2_ atmosphere is an essential driving force for the dispersion of nearly-metallic Ag nanoclusters under in situ conditions, verified by both experimental evidence and density functional theory (DFT) calculations. The dynamic dispersion of Ag during the reaction and its correlation to the catalytic reactivity is further demonstrated for CO oxidation reaction.

## Results

### Ex situ characterization of oxidation-induced dispersion of AgNWs

**Fig. 1:**
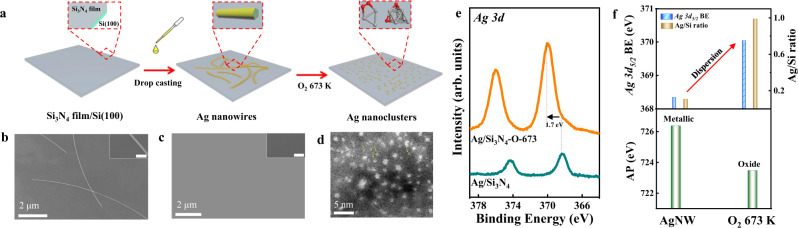
Oxidative dispersion of AgNWs. **a** Scheme for the preparation of AgNWs onto Si_3_N_4_ (Ag/Si_3_N_4_) and the subsequent oxidation treatment (Ag/Si_3_N_4_-O-673). **b**, **c** SEM images of Ag/Si_3_N_4_ and Ag/Si_3_N_4_-O-673 samples. The scale bar in the insets is 200 nm. **d** HAADF-STEM image of Ag/Si_3_N_4_-O-673. **e** XPS Ag 3*d* spectra and **f** Ag 3*d*_5/2_ BE shift, Ag/Si ratio, and Auger parameter deduced from quasi in situ XPS data of Ag/Si_3_N_4_ and Ag/Si_3_N_4_-O-673 samples.

Well-defined AgNWs^32^ were drop-casted onto a Si_3_N_4_ thin film (50 nm thick) grown on a Si(100) wafer, denoted as Ag/Si_3_N_4_ (see “Methods” and Fig. 1a). The as-prepared AgNWs (Fig. 1b) show an average length of 20 μm with 80 nm in diameter. Thermal treatment in 1 mbar O_2_ at 673 K results in the dispersion of all AgNWs into ultra-small Ag clusters on the Si_3_N_4_ surface (denoted as Ag/Si_3_N_4_-O-673), rather than sintered aggregates. The small size makes them nearly invisible under SEM imaging as shown by Fig. 1c. High angle annular dark-field (HAADF)-STEM imaging further reveals the highly dispersed sub-nm Ag nanoclusters (Ag_*n*_) with a significant number of atomic clusters and single atoms (marked by yellow arrows in Fig. 1d and supplementary Fig. 1).

Measured by quasi in situ XPS (Fig. 1e), the pristine Ag/Si_3_N_4_ surface shows Ag 3*d*_5/2_ binding energy (BE) of 368.3 eV which is characteristic of metallic silver^33^, whereas the Ag/Si_3_N_4_-O-673 sample presents a significant upshift of Ag 3*d*_5/2_ by +1.7 eV to 370.0 eV. Both Si 2*p* and N 1*s* spectra of the two samples remain unchanged (Supplementary Fig. 2), suggesting the high stability of the Si_3_N_4_ film under the treatment condition. It is well known that Ag oxides usually have a similar or even lower Ag 3*d* BE compared to Ag metal^12^. Therefore, the upshift of Ag 3*d* BE in Ag/Si_3_N_4_-O-673 is not caused by the change of oxidation state, but more likely due to size effect^34,35^. It has been validated that the screening of core holes in ultra-small metal nanoclusters becomes limited which results in an upshift of core-level BEs^36–39^. Hence, the upshift of Ag 3*d* BE is ascribed to the decreasing size of Ag aggregates from micrometer to sub-nanometer. Meanwhile, considering the surface sensitivity of XPS, Ag 3*d* peak intensity of the Ag/Si_3_N_4_-O-673 sample also increases drastically, reflecting the gain of Ag surface area (surface-to-bulk ratio) during the transformation of AgNWs to highly dispersed Ag nanoclusters. To quantify, the peak area ratio of Ag 3*d*_5/2_ to Si 2*p* (denoted as Ag/Si ratio) was derived and used as a representative for the dispersion degree of Ag nanostructures on Si_3_N_4_. As illustrated in Fig. 1f, both Ag/Si ratio and Ag 3*d*_5/2_ BE reflect the Ag dispersion degree for a given coverage (referred to Ag loadings on Si_3_N_4_ substrate), suggesting the high dispersion of AgNWs after the O_2_ treatment as proved by the SEM/STEM results (Fig. 1b–d).

Regarding the oxidation state of silver, it is unable to distinguish oxide or metallic state using XPS core-levels^12,33^. Alternatively, Auger Parameters (AP, α) (see the description of Auger parameter in the Supplementary Discussion) defined as the sums of Auger kinetic energy (KE) and photoelectron BE are often used to identify the chemical state of elements including Cu, Ag, Zn, etc^37,40,41^. Auger spectra of Ag MNN are shown in supplementary Fig. 2, and the sums of Ag 3*d*_5/2_ BE and Ag MNN KE were given in Fig. 1f. AP value for pristine Ag/Si_3_N_4_ is about 726.4 eV as characteristic for metallic Ag^12,40^, while that of Ag/Si_3_N_4_-O-673 shows a lower value of 723.6 eV reflecting its nature of Ag oxides (e.g., Ag_2_O)^12^ after the O_2_ treatment. Overall, we can thus establish a criterion by using Ag/Si ratio and Ag 3*d*_5/2_ BE as indications for Ag dispersion degree, and AP value to describe the oxidation state. To further validate the universality of this dynamic dispersion of Ag on different supports, we have performed additional experiments on a variety of oxides including SiO_2_/Si(100), Al_2_O_3_(0001), and SrTiO_3_(110) substrates. The upshift of Ag 3*d* BE and the increase in the Ag 3*d* peak intensity were observed on all the substrates (supplementary Fig. 3), which is similar to that on the Si_3_N_4_ surface (Supplementary Discussion).

### Reversible oscillation of Ag dispersion upon cyclic oxidation–reduction treatments

**Fig. 2:**
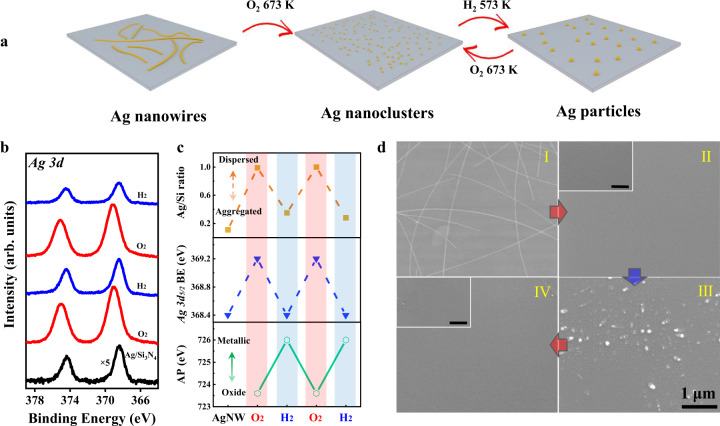
Reversible oscillation of Ag dispersion during redox cycles. **a** Schemes of redox cycles of Ag aggregates on Si_3_N_4_. **b** Quasi in situ XPS Ag 3*d* spectra of Ag nanostructures on Si_3_N_4_ during redox cycles. **c** Oscillation of Ag/Si ratio, Ag 3*d*_5/2_ BE, and Auger parameter during O_2_/H_2_ redox cycles. **d** Ex situ SEM images of Ag/Si_3_N_4_ sample upon repeated O_2_/H_2_ redox treatment. The scale bar in the insets is 500 nm. I: pristine Ag/Si_3_N_4_ sample; II: oxidation in 1 bar O_2_ at 673 K; III: subsequent reduction in 0.1 bar H_2_ at 573 K; IV: re-oxidation in 1 bar O_2_ at 673 K. All XPS spectra were collected at room temperature.

Cyclic oxidation–reduction treatments (Fig. 2a) were performed to investigate the dispersion-aggregation of Ag nanostructures in an alternative O_2_ and H_2_ atmosphere. Quasi in situ Ag 3*d* XPS spectra (Fig. 2b) and Ag MNN Auger spectra (supplementary Fig. 4) as well as ex situ SEM images (Fig. 2d) reveal good reversibility of Ag dispersion upon the oxidation–reduction cycles. The deduced Ag/Si ratio, Ag 3*d*_5/2_ BE, and AP values are presented in Fig. 2c, and an oscillation of Ag dispersion and oxidation states is clearly demonstrated. After the first oxidation treatment (1 bar O_2_ at 673 K for 1 h), a highly dispersed state of silver oxide (AgO_*x*_) nanoclusters was identified at room temperature. The increasing Ag/Si ratio and the upshift of Ag 3*d*_*5/2*_ BE are characteristic of the high dispersion state, and the lower AP value infers its oxide nature. The ultra-small AgO_*x*_ nanoclusters are likely below 1 nm making them invisible under SEM imaging (Fig. 2d, II).

The subsequent reduction (0.1 bar H_2_ at 573 K for 1 h) leads to strong sintering as indicated by the decreasing Ag/Si ratio and the downshift of Ag 3*d*_*5/2*_ BE. The reduction to metallic Ag state is verified by the higher AP value. The sintered Ag metal particles show a size range of 10–200 nm measured by SEM (Fig. 2d, III). The re-oxidation (Fig. 2d, IV) can repeatedly disassemble the aggregated Ag particles into ultra-small AgO_*x*_ clusters. The results indicate that the oxidative dispersion behavior does not rely on the morphology of Ag aggregates, no matter of NWs or NPs. The second reduction shows a good reproducibility yielding metallic Ag aggregates after the H_2_ treatment. The observed reversible oscillation of Ag dispersion during redox cycles opens the feasibility to control the dispersion of Ag catalysts via oxidation/reduction pretreatments.

Solely based on quasi in situ XPS and ex situ EM results, we can demonstrate oxidative dispersion of μm-scale metallic AgNWs into sub-nm AgO_*x*_ clusters through 1 mbar–1 bar O_2_ treatment at 673 K. These ex situ observations are seemingly in line with the previous understanding for the oxidation-induced dispersion mechanism. However, our in situ experiments disproved this “common sense” and further revealed the role of oxygen atmosphere as discussed in the following section.

### In situ observation of oxidative dispersion

**Fig. 3:**
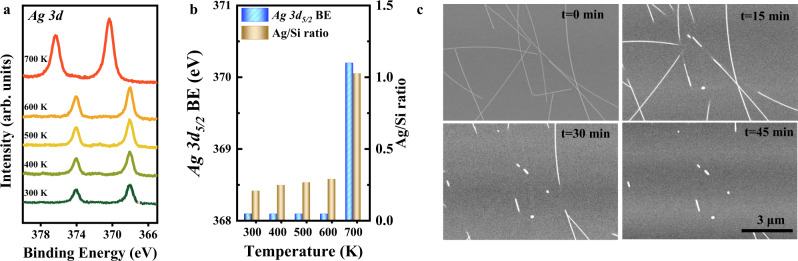
In situ observation of dynamic dispersion of AgNWs in 1 mbar O_2_. **a** NAP-XPS Ag 3*d* spectra and **b** Ag 3*d*_5/2_ BE and Ag/Si ratio of Ag/Si_3_N_4_ sample upon heating from 300 to 700 K in 1 mbar O_2_. **c** In situ visualization of dynamic dispersion of AgNWs at 673 K in 1 mbar O_2_ by ESEM.

In order to elucidate the dynamic process of the Ag dispersion, we performed NAP-XPS studies on Ag/Si_3_N_4_ in 1 mbar O_2_ during heating from 300 to 700 K (Fig. 3a). No obvious change in XPS Ag 3*d* spectra was observed in a range of 300–600 K. The slight increase of Ag 3*d* peak intensity is ascribed to the removal of carbon residuals on the sample surface (see C 1*s* spectra in Supplementary Fig. 5). Further increasing temperature to 700 K results in a sharp increase of Ag/Si ratio along with a prominent BE shift of Ag 3*d*_5/2_ from 368.1 to 370.2 eV (Fig. 3b), which serves as a clear indication for the dispersion of AgNWs into small Ag clusters.

Direct imaging of dynamic dispersion of AgNWs was performed in situ using ESEM in 1 mbar O_2_ at 673 K (Fig. 3c and Supplementary Video 1). Initially, intact AgNWs were observed on Si_3_N_4_ surface at *t* = 0 min. Subsequently, the dispersion of AgNWs occurs at *t* = 15 min, along with the spread-out of Ag nanoclusters (<1 nm) to the free substrate surface resulting in the invisibility of Ag under SEM (*t* = 30 min). The dispersion sustains with increasing time and eventually leads to a nearly complete disappearance of AgNWs at *t* = 45 min. Similarly, the dispersion process was further confirmed by NAP-PEEM^42^ (Supplementary Fig. 6), which was conducted at 873 K in O_2_ ambience up to 0.1 mbar. The bright contrast of AgNWs in PEEM images is due to the low surface work function of metallic Ag^43^. When exposed to O_2_, part of NW was observed to become dark and the darkening gradually extended to the whole NW (Supplementary Fig. 6), indicating the diffusion of silver atoms from NWs to the bared substrate surface^43^. Both in situ spectroscopic and microscopic measurements provide a clear picture of the high dispersion of AgNWs at elevated temperatures driven by O_2_.

### Role of oxygen: surface oxygen adsorption vs. bulk oxidation

**Fig. 4:**
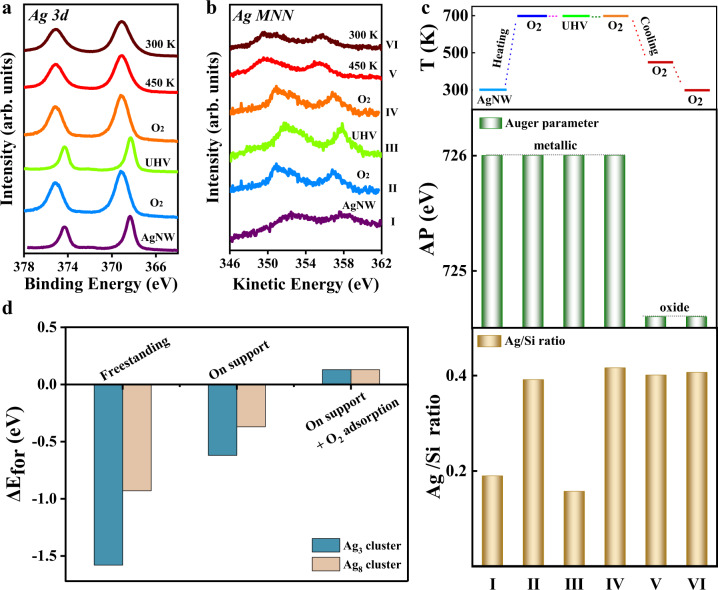
Evolution of Ag during in situ experimental conditions. NAP-XPS results of Ag 3*d* spectra (**a**) and Ag MNN Auger spectra (**b**) of Ag/Si_3_N_4_ under in situ experimental conditions. I: Ag/Si_3_N_4_; II: 1 mbar O_2_ at 700 K; III: UHV (1 × 10^−^^6^ mbar) at 700 K; IV: 1 mbar O_2_ at 700 K; V: cooling to 450 K in 1 mbar O_2_; VI: cooling to 300 K in 1 mbar O_2_. **c** Deduced Auger parameter and Ag/Si ratio from NAP-XPS data, and temperature profile for in situ experimental conditions. **d** The relative formation energy of Ag_3_ cluster and Ag_8_ cluster based on the Ag particle under considering different influencing factors.

It has been well accepted that bulk oxidation with a high oxidation degree is the key step in the dispersion of metal particles in O_2_ at elevated temperatures^6,11,14,20,44,45^. However, whether bulk oxidation is necessary for oxidative dispersion of supported metal catalysts still requires to be validated. To this end, we performed NAP-XPS studies of AgNWs on Si_3_N_4_ under different conditions in the following sequence: I, pristine Ag/Si_3_N_4_ under UHV at room temperature; II, 1 mbar O_2_ at 700 K; III, UHV at 700 K; IV, 1 mbar O_2_ at 700 K; V, 1 mbar O_2_ at 450 K; VI, 1 mbar O_2_ at 300 K. All XPS core-level and Auger peaks were measured in situ at the treatment temperatures and under the certain environments as presented in Fig. 4a, b, and the corresponding Ag/Si ratios and AP values were displayed in Fig. 4c.

The results show that calcinating at 700 K in 1 mbar O_2_ (condition II) leads to a significant increase in Ag/Si ratio indicating the dispersion of AgNWs. The AP value remains at 726.0 eV (errors in the range from −0.3 to 0.3 eV due to the broad Ag Auger peaks) that strongly infers a metallic state of dispersed Ag species under in situ condition (700 K, 1 mbar O_2_). This can be rationalized by the Ag-O phase diagram showing that oxides are prone to decompose into metallic phase at 700 K in 1 mbar O_2_^29^. It suggests that the formation of bulk oxide with a high oxidation degree is not necessary for stabilizing highly dispersed metallic species. To our best knowledge, it is the first evidence that elucidates the dispersion in the form of nearly-metallic clusters rather than bulk oxide (with a high oxidation degree) during oxidative dispersion. A similar metallic state of dispersed Ag species was also observed on the SiO_2_/Si(100) surface in O_2_ at elevated temperatures, verifying a universal phenomenon for SiO_2_ and other oxides (Supplementary Fig. 7).

After removing mbar O_2_ to UHV condition (condition III), sintering of dispersed Ag nanoclusters occurs, forming aggregated particles as indicated by the smaller Ag/Si ratio. In absence of O_2_ atmosphere, the gain of surface energy favors the sintering of Ag metal particles, e.g., Ostwald ripening, which suggests that adsorbed oxygen species from the O_2_ atmosphere play a critical role in stabilizing highly dispersed Ag nanoclusters. Previous surface science studies on Ag foils and Ag single crystals indicated that adsorbed oxygen species are formed at Ag surfaces at high temperature in O_2_^46–48^, rather than producing bulk oxides (Ag_2_O). Hence, chemisorption of oxygen on metallic Ag surface is suggested as a possible driving force for the dynamic dispersion of AgNWs/aggregates into sub-nm Ag clusters during the oxidative dispersion. The reversibility of adsorption-driven dispersion-aggregation is further proved by re-exposure of the sintered Ag aggregates to O_2_ at 700 K (Fig. 4c IV). During the entire process, nearly-metallic state of Ag is preserved as indicated by the constant AP value of 726.0 eV for all four states (I, II, III, and IV).

The cooling process of highly dispersed Ag nanoclusters in 1 mbar O_2_ from 700 to 300 K was further investigated by changing from condition IV to VI. Cooling down in 1 mbar O_2_ to 450 and 300 K results in a prominent downshift of AP value to 724.6 eV suggesting the formation of AgO_*x*_ upon cooling in O_2_ (Fig. 4c, IV–VI). Meanwhile, the constant Ag/Si ratio (Fig. 4c, IV–VI) reveals that the high dispersion of Ag nanoclusters is still preserved at low temperatures. Therefore, we can depict an overall picture of dynamic dispersion of AgNWs in 1 mbar O_2_ by two independent processes: (1) surface oxygen adsorption-driven dispersion of AgNWs to Ag nanoclusters at 700 K, and (2) oxidation of readily dispersed Ag nanoclusters to bulk-like AgO_*x*_ nanoclusters with a high oxidation degree during cooling to 300 K. Our in situ characterizations allow to conclude that the chemisorption of oxygen on metallic silver is the driving force for Ag dispersion whereas the bulk oxide with a high oxidation degree is only the result upon cooling in O_2_.

### DFT study in oxidative dispersion mechanism

We proposed a DFT-based model to understand the O_2_ adsorption-induced dispersion. The equation for this model is described in the “Methods” section (Eq. (1)). The competing mechanism among cohesive energy (*E*_coh_, metal-metal interaction), adhesion energy (*E*_adh_, metal-support interaction), and gas adsorption energy (*E*_ads_, metal-gas interaction) has been considered thoroughly, the sum of which constitutes the formation energy of the NP/cluster on the support under in situ conditions. The difference of the averaged formation energy/atom between the sintered NP and the dispersed cluster $({\Delta}E_{\mathrm{for}})$, thus determines the dynamic dispersion, and the positive ${\Delta}E_{\mathrm{for}}$ favors the dispersion.

Considering the very large average sizes of the sintered NPs in this work (over 100 nm), we used the cohesive energy/atom of bulk Ag $(E_{\mathrm{{coh}}}^{\mathrm{{bulk}}})$ to represent the NP’s *E*_coh_ (set *α* in Eq. (1) to be 12), which is −2.79 eV in our calculation. Ag_3_ and Ag_8_ clusters were used as the models for the cluster calculations, the $E_{\mathrm{{coh}}}^{\mathrm{{clu}}}$ of which are calculated to be −1.21 and −1.86 eV, respectively. Thus, in their freestanding states, ΔE_for_ are −1.58 and −0.93 eV, respectively (Fig. 4d). When supported on the surface, the averaged *E*_adh_/atom of the sintered NP is neglectable because the ratio of the interface to volume for such a large particle is very small. On the other hand, the averaged *E*_adh_/atom of Ag_3_ and Ag_8_ clusters are calculated to be −0.96 and −0.56 eV on the supported Si_3_N_4_(0001) surface (Supplementary Figs. 8, 9), respectively. Consequently, ${\Delta}E_{\mathrm{for}}$ becomes −0.62 and −0.37 eV, respectively, which are still negative, suggesting that the addition of metal-support interaction is insufficient to disperse Ag NPs into nanoclusters. This thus explains the aggregated state of Ag particles under UHV condition during in situ experiments in Fig. 4c, III when O_2_ is removed from the atmosphere.

When O_2_ adsorption is involved, the reaction condition effects (temperature and gas pressure) are considered through the calculation of gas adsorption coverage *θ*^*i*^ by using a DFT-based thermodynamic adsorption isotherm described in the methods section^49–51^. We used the gas coverage of O_2_ on three low-index surfaces ($\theta ^{111},\;\theta ^{110},\;\theta ^{100}$) to estimate *θ*^*i*^ of the sintered NP because a nanosized Ag particle is normally composed of these surfaces (supplementary Fig. 10). The results show that O_2_ barely adsorbs on the three surfaces at 673 K even with an O_2_ pressure of 1 bar due to the small adsorption energies ($E_{\mathrm{ads}}^{111} = - 0.40$ eV, $E_{\mathrm{ads}}^{110} = - 0.38$ eV, $E_{\mathrm{ads}}^{100} = - 0.76$ eV). On the contrary, associative O_2_ and dissociative O atoms adsorb stably at the Ag_3_-Si_3_N_4_ ($E_{\mathrm{ads}}^{\mathrm{clu}} = - 2.24\;{\mathrm{eV}}$) and Ag_8_-Si_3_N_4_ ($E_{\mathrm{ads}}^{\mathrm{clu}} = - 3.97\;{\mathrm{eV}}$) interfaces, which leads to $\theta ^{\mathrm{clu}} = 1$ for both models at 673 K under either 1 mbar O_2_ or 1 bar O_2_. The averaged $E_{\mathrm{ads}}^{\mathrm{clu}}$/atom is −0.75 and −0.50 eV, respectively, which eventually makes ${\Delta}E_{\mathrm{for}}$ positive (0.13 eV for both models, Fig. 4d). Compared to the sintered NP, dispersed nanoclusters have more contact with the support surface and the O_2_ gas environment. Neither the support effect nor the O_2_ adsorption could cause the dispersion of Ag NPs observed in our experiments, but the combined effects are enough to pry the lever.

### Dynamic dispersion during CO oxidation reaction

**Fig. 5:**
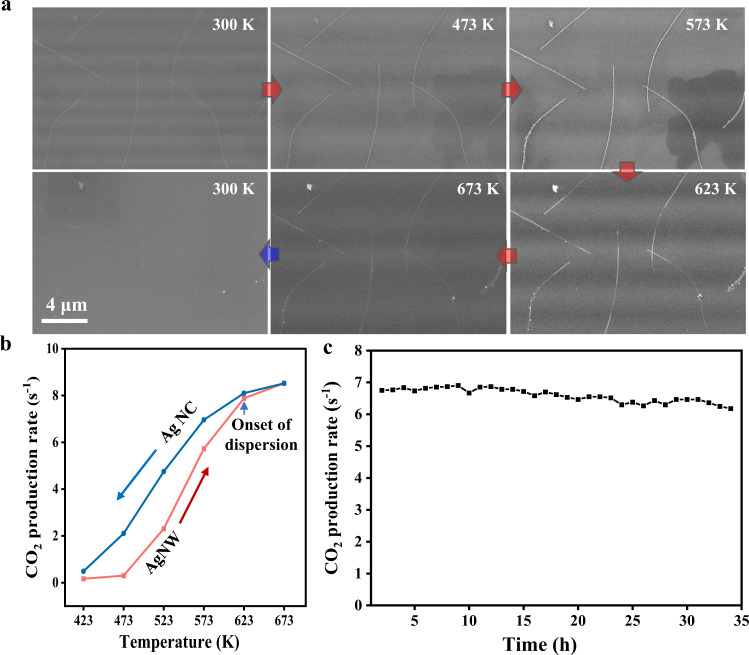
Dynamic Ag dispersion during CO oxidation. **a** In situ ESEM imaging of dynamic Ag dispersion during the reaction in 5% CO/95% O_2_ at 1 mbar. The scale bar is 4 μm for all ESEM images. **b** The CO conversion for Ag/Si_3_N_4_ upon the heating and cooling cycle. **c** The time-on-stream stability test of Ag/Si_3_N_4_ under the reaction condition at 673 K.

The dynamic dispersion of Ag nanostructures during the reaction was further verified for CO oxidation^11,48,52–54^ and the impact on their catalytic activity was investigated. In situ ESEM images (Fig. 5a) show the dynamic dispersion of AgNWs under reaction conditions of 5% CO/95% O_2_ at 1 mbar. The dispersion of AgNWs initially occurs at 623 K and reaches a highly dispersed state at 673 K. The highly dispersed Ag nanoclusters remain in the reaction gases during cooling. The catalytic test was further performed in a home-built flow reactor with an online mass spectrometer to the outlet for product analysis (see Supplementary Methods). The reactivity for catalytic CO oxidation reaction was present in Fig. 5b and Supplementary Fig. 11, and a hysteresis phenomenon was clearly identified upon heating and cooling cycle, as the result of the dynamic structure evolution of Ag during the reaction. The highly dispersed Ag nanoclusters formed during reaction exhibit an enhanced activity especially at low temperature, higher than the initial state of AgNWs upon heating. A similar reactivity at 623 K indicates the structural transformation from NWs to nanoclusters which is consistent with the in situ ESEM observations. The time-on-stream stability test displayed in Fig. 5c further reveals excellent durability of the highly dispersed Ag/Si_3_N_4_ catalyst under the reaction condition (at 673 K) up to 35 h (also see Supplementary Discussion). Our results thus prove the long-term stability of dispersed nearly-metallic Ag nanoclusters in O_2_-rich reaction environments, and opens up a new route for the fabrication of highly dispersed catalysts during the reaction.

## Discussion

**Fig. 6:**
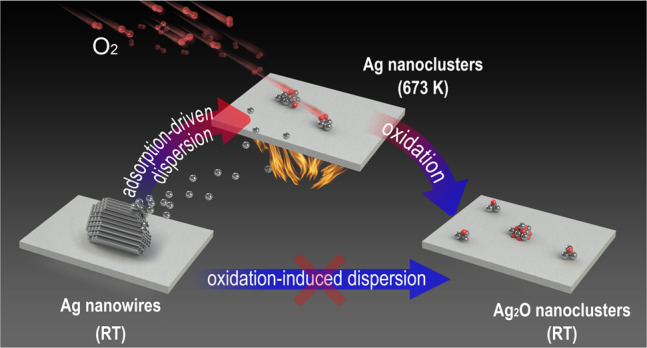
Dynamic evolution of Ag during oxidative dispersion. The description of adsorption-driven dispersion mechanism rather than oxidation-induced dispersion mechanism.

Dynamic dispersion of AgNWs into sub-nm Ag clusters on Si_3_N_4_ surface has been observed using combined ex situ and in situ characterization techniques. By altering oxidizing/reducing treatments, we can reversibly switch between highly dispersed Ag nanoclusters (0.7 nm) and aggregated Ag particles (20–100 nm). Based on ex situ characterizations, we readily observed the formation of dispersed silver oxides at room temperature after the high-temperature treatment in O_2_, which is in line with the commonly accepted “oxidation dispersion” scenario. However, as Fig. 6 summarized, our in situ studies directly demonstrated a transitional state of nearly-metallic Ag during dispersion that overturn this common concept and depict an overall scenario of dynamic dispersion under O_2_ environment consisting of oxygen adsorption-induced dispersion of Ag in a nearly-metallic state at 700 K and oxidation of readily dispersed Ag nanoclusters to Ag oxide nanoclusters during cooling to 300 K. We provide solid evidence for the fact that formation of bulk oxide with a high oxidation degree is not necessary in oxidative dispersion, but a result during cooling in O_2_. Chemisorption of oxygen on nearly-metallic Ag rather than bulk oxidation is proved as the driving force to disperse Ag aggregates and stabilize ultra-small Ag clusters under in situ conditions. Correctly understanding the role of O_2_ environment played in oxidative dispersion is of great significance in predicting and controlling the dynamics of dispersion/redispersion of supported metal catalysts under reaction conditions. It also opens up a new route to stabilize metal nanoclusters in a realistic reaction atmosphere, and benefits the fabrication of highly/atomically dispersed catalyst during reaction.

## Methods

### Sample preparation and treatment

AgNWs were synthesized via a polyol method and then purified by a novel dynamic agitation-induced centrifugal filtration^32^. Si_3_N_4_ film (50-nm thick) grown on Si(100) single crystals were purchased from Prmat (Shanghai) Technology Co., Ltd. The inert Si_3_N_4_ films are nearly free of surface defects^55^ and remain stable under reaction conditions (see Supplementary Discussion). AgNWs in ethanol solution were drop-casted onto the Si_3_N_4_ substrate (denoted as Ag/Si_3_N_4_). We prepared three kinds of samples with different Ag densities (high, medium, and low). To acquire strong Auger spectra, samples with high NW density were used and shown in Fig. 4. Samples with medium AgNW density were used to get an obvious change of Ag/Si ratio during various treatments, also to make XPS peak intensity strong enough, shown in Figs. 1–3. Samples with low density were used in electron microscopy (SEM, STEM, PEEM) to observe the process easily. The effect of surface concentration in our experiments was excluded by SEM results (Supplementary Fig. 16). Ex situ treatments were performed in 1 mbar O_2_, 1 bar O_2_, or 100 mbar H_2_ at elevated temperatures in a high-pressure reactor (SPECS, HPC-20) (denoted as Ag/Si_3_N_4_-*x*-*y*, *x* is the atmosphere and *y* is the temperature).

### Scanning transmission electron microscopy

AC-STEM was performed on a JEM ARM200F (JEOL, Japan), equipped with a thermal-field emission gun and a Cs-corrector. The microscope was operated at 80 kV to minimize the beam damage. For the high angle annular dark-field (HAADF) imaging, the convergence angle of ~23 mrad and collection angle range of 68–174 mrad were adapted for the incoherent atomic number imaging.

### In situ NAP-XPS

Surface chemistry of Ag/Si_3_N_4_ was characterized in a lab-based near-ambient pressure X-ray photoelectron spectroscopy (SPECS EnviroESCA). Spectra were obtained using monochromatic Al Kα irradiation (1486.7 eV) of an Al anode operated at 50 W. A pass energy of 40 eV with a step of 0.05 eV and a dwell time of 0.1 s were typically used for acquiring the core-level spectra.

### Quasi in situ XPS

Quasi in situ XPS measurements were carried out with a spectrometer equipped with an Mg Kα X-ray source operated at 300 W. The background pressure was in the range of 10^−9^ mbar. Ex situ treatments were performed in the high-pressure reactor (SPECS, HPC-20). After each treatment, the sample was transferred to the analysis chamber for XPS measurements without exposure to air. All quasi in situ XPS spectra were collected under UHV (10^−9^–10^−^^10^ mbar) at room temperature.

### In situ ESEM

In situ SEM imaging was performed in a commercial ESEM (FEI Quanta 650) equipped with a commercial heating stage and a gas feeding unit. The accelerating voltage was 20 kV with an objective lens aperture of 30 μm. During heating, low magnification was used to locate the targeted area due to thermal drift, and high magnification imaging (×20,000) was conducted when the temperature is stable.

### In situ NAP-PEEM

PEEM experiments were performed on a newly developed NAP-PEEM system, which enables high-resolution PEEM imaging in gases with pressures up to 1 mbar. A tunable deep ultraviolet (DUV) laser with wavelength between 175 and 210 nm was used as an excitation source. The sample was heated up by a specially design laser heater, and the sample temperature was measured by an infrared thermometer. The in situ PEEM imaging was conducted by first heating the sample to a specific temperature and then dosing O_2_ with increasing pressures.

### Catalytic testing

The sample was prepared by drop-casting of AgNWs onto Si_3_N_4_/Si(100) surfaces (10 mm × 10 mm) with a silver loading of 1 μg. The catalytic test was performed in a home-built cell reactor (setup details see Supplementary Methods) in a gas flow of 10 ml/min 5% CO/95% O_2_ at atmospheric pressure. The reaction product (CO_2_, *m*/*z* 44) was monitored by an online mass spectrometer (SRS RGA) to the outlet of the reactor. The cell reactor was repeatedly purged and flushed with the reaction gas for >6 h to achieve a clean background. All catalysts were pre-reduced in 5% H_2_ at 100 °C for 30 min before catalytic measurements.

### Equation for the adsorption-induced dispersion model

Equation (1) is the formula we used to estimate the energy difference per atom between a sintered NP and dispersed clusters on the support under the O_2_ gas conditions.

1 $${\Delta}E_{\mathrm{for}} = \frac{\alpha }{{12}}E_{\mathrm{coh}}^{\mathrm{bulk}} + \left( {\frac{{E_{\mathrm{adh}}^{\mathrm{par}}}}{{N_{\mathrm{par}}}} + \frac{{\mathop {\sum }\nolimits_{i = 1}^{N_{\mathrm{surf}}} \theta ^iE_{\mathrm{ads}}^i}}{{N_{\mathrm{par}}}}} \right) - E_{\mathrm{coh}}^{\mathrm{clu}} - \left( {\frac{{E_{\mathrm{adh}}^{\mathrm{clu}}}}{{N_{\mathrm{clu}}}} + \frac{{\theta ^{\mathrm{clu}}E_{\mathrm{ads}}^{\mathrm{clu}}}}{{N_{\mathrm{clu}}}}} \right)$$

The first term in Eq. (1) is the cohesive energy per atom of the sintered NP, in which $E_{\mathrm{coh}}^{\mathrm{bulk}}$ is the cohesive energy of a bulk Ag atom, *α* represents for the average coordination number of a sintered NP, and 12 is the coordination number of bulk Ag. The support effect and the O_2_ adsorption effect on the sintered NP are considered in the second term, in which $E_{\mathrm{adh}}^{\mathrm{par}}$ is the adhesion energy of the NP on the support, *θ*^*i*^ is the adsorption coverage of O_2_ at the *i*th surface site, $E_{\mathrm{ads}}^i$ is the corresponding adsorption energy, $N_{\mathrm{surf}}$ is the number of surface sites, and $N_{\mathrm{par}}$ is the number of atoms of the NP. The third term ($E_{\mathrm{coh}}^{\mathrm{clu}}$) is the cohesive energy of the dispersed cluster averaged to each atom. The support effect and the O_2_ adsorption effect on the dispersed cluster are considered in the fourth term, in which $E_{\mathrm{adh}}^{\mathrm{clu}}$ is the adhesion energy of the cluster on the support, $\theta ^{\mathrm{clu}}$ is the adsorption possibility, $E_{\mathrm{ads}}^{\mathrm{clu}}$ is the corresponding adsorption energy, and $N_{\mathrm{clu}}$ is the number of atoms of the cluster. If the formation energy difference per atom ${\Delta}E_{\mathrm{for}}$ is larger than zero, the dispersion of the nanoclusters is preferred. The DFT settings can be found in the Supplementary Information.

### DFT-based thermodynamic adsorption isotherm

The O_2_ coverage (*θ*) at a given adsorption site was described using the Langmuir adsorption isotherm. The formula is shown below,

2 $$\theta = PK/(1 + PK)$$

where *P* is the gas pressure, *K* is the equilibrium constant calculated as below:

3 $$K = {\mathrm{exp}}\left( { - \frac{{{\Delta}G}}{{RT}}} \right) = {\mathrm{exp}}\left( { - \frac{{E_{\mathrm{ads}} - T\left( {S_{\mathrm{ads}} - S_{\mathrm{gas}}} \right)}}{{RT}}} \right)$$

where *R* is the gas constant, *T* is the temperature, $E_{\mathrm{ads}}$ is the gas adsorption energy obtained from DFT calculations, $S_{\mathrm{ads}}$ and $S_{\mathrm{gas}}$ are the entropy of adsorbed O_2_ and gas-phase O_2_, respectively. In this work, $S_{\mathrm{ads}}$ was set to be 0 and $S_{\mathrm{gas}}$ was obtained from the thermodynamic table (https://www.nist.gov/srd).

## Supplementary information

Supplementary Information

Peer Review File

Description of Additional Supplementary Files

Supplementary Movie 1

## Data Availability

The data that support the plots within this paper and other findings of this study are available from the corresponding author upon reasonable request due to the data are of large amount.

## References

[1] Campbell CT, Parker SC, Starr DE (2002). The effect of size-dependent nanoparticle energetics on catalyst sintering. Science.

[2] Argyle MD, Bartholomew CH (2015). Heterogeneous catalyst deactivation and regeneration: A review. Catalysts.

[3] Hansen TW, Delariva AT, Challa SR, Datye AK (2013). Sintering of catalytic nanoparticles: Particle migration or ostwald ripening?. Acc. Chem. Res..

[4] Morgan K, Goguet A, Hardacre C (2015). Metal redispersion strategies for recycling of supported metal catalysts: a perspective. ACS Catal..

[5] Sa J (2012). Redispersion of gold supported on oxides. ACS Catal..

[6] Moliner M (2016). Reversible transformation of Pt nanoparticles into single atoms inside high-silica chabazite zeolite. J. Am. Chem. Soc..

[7] Birgerssona H, Boutonnet M, Lars ESJ (2004). Deactivation and regeneration of spent three-way automotive exhaust gas catalysts (TWC). Top. Catal..

[8] Nishihata Y (2002). Self-regeneration of a Pd-perovskite catalyst for automotive emissions control. Nature.

[9] Ganzler AM (2017). Tuning the structure of platinum particles on ceria in situ for enhancing the catalytic performance of exhaust gas catalysts. Angew. Chem. Int. Ed..

[10] Dessal C (2019). Atmosphere-dependent stability and mobility of catalytic Pt single atoms and clusters on γ-Al_2_O_3_. Nanoscale.

[11] Zhang Z (2017). Thermally stable single atom Pt/m-Al_2_O_3_ for selective hydrogenation and CO oxidation. Nat. Commun..

[12] Huang Z (2012). Catalytically active single-atom sites fabricated from silver particles. Angew. Chem. Int. Ed..

[13] Jeong H (2018). Fully dispersed Rh ensemble catalyst to enhance low-temperature activity. J. Am. Chem. Soc..

[14] Spezzati G (2017). Atomically dispersed Pd-O Species on CeO_2_(111) as highly active sites for low-temperature CO oxidation. ACS Catal..

[15] Wang A, Li J, Zhang T (2018). Heterogeneous single-atom catalysis. Nat. Rev. Chem..

[16] Jones J (2016). Thermally stable single-atom platinum-on-ceria catalysts via atom trapping. Science.

[17] Wang F (2020). Resolving the puzzle of single-atom silver dispersion on nanosized γ-Al_2_O_3_ surface for high catalytic performance. Nat. Commun..

[18] Liu K (2020). Strong metal-support interaction promoted scalable production of thermally stable single-atom catalysts. Nat. Commun..

[19] Lang R (2019). Non defect-stabilized thermally stable single-atom catalyst. Nat. Commun..

[20] Aitbekova A (2018). Low-temperature restructuring of CeO_2_-supported Ru nanoparticles determines selectivity in CO_2_ catalytic reduction. J. Am. Chem. Soc..

[21] Maurer F (2020). Tracking the formation, fate and consequence for catalytic activity of Pt single sites on CeO_2_. Nat. Catal..

[22] Nagai Y (2008). In situ redispersion of platinum autoexhaust catalysts: an on-line approach to increasing catalyst lifetimes?. Angew. Chem. Int. Ed..

[23] Ferré G (2020). Exploiting the dynamic properties of Pt on ceria for low-temperature CO oxidation. Catal. Sci. Technol..

[24] DeRita L (2019). Structural evolution of atomically dispersed Pt catalysts dictates reactivity. Nat. Mater..

[25] Kibis LS (2017). Redox and catalytic properties of Rh_x_Ce_1–x_O_2−δ_ solid solution. J. Phys. Chem. C.

[26] Gao Y (2017). Aggregation and redispersion of silver species on alumina and sulphated alumina supports for soot oxidation. Catal. Sci. Technol..

[27] Gardini D, Christensen JM, Damsgaard CD, Jensen AD, Wagner JB (2016). Visualizing the mobility of silver during catalytic soot oxidation. Appl. Catal. B.

[28] Ikemoto S (2019). Reversible low-temperature redox activity and selective oxidation catalysis derived from the concerted activation of multiple metal species on Cr and Rh-incorporated ceria catalysts. Phys. Chem. Chem. Phys..

[29] Li W, Stampfl C, Scheffler M (2003). Why is a noble metal catalytically active? The role of the O-Ag interaction in the function of silver as an oxidation catalyst. Phys. Rev. Lett..

[30] Ketteler G (2005). In situ spectroscopic study of the oxidation and reduction of Pd(111). J. Am. Chem. Soc..

[31] Tsai HC (2003). Instability of gold oxide Au_2_O_3_. Surf. Sci..

[32] Wang H, Tang H, Liang J, Chen Y (2018). Dynamic agitation-induced centrifugal purification of nanowires enabling transparent electrodes with 99.2% transmittance. Adv. Funct. Mater..

[33] Schon G (1973). ESCA studies of Ag, Ag_2_O and AgO. Acta Chem. Scand..

[34] Luo K, Clair TPS, Lai X, Goodman DW (2000). Silver growth on TiO_2_(110) (1 × 1) and (1 × 2). J. Phys. Chem. B.

[35] Guo D, Guo Q, Zheng K, Wang E, Bao X (2007). Initial growth and oxygen adsorption of silver on Al_2_O_3_ film. J. Phys. Chem. C.

[36] Mao B (2014). A near ambient pressure XPS study of subnanometer silver clusters on Al_2_O_3_ and TiO_2_ ultrathin film supports. Phys. Chem. Chem. Phys..

[37] Moretti G (2013). The Wagner plot and the Auger parameter as tools to separate initial- and final-state contributions in X-ray photoemission spectroscopy. Surf. Sci..

[38] Bagus PS, Wieckowski A, Freund H (2006). Initial and final state contributions to binding-energy shifts due to lattice strain: validation of Auger parameter analyses. Chem. Phys. Lett..

[39] Fu Q, Wagner T (2007). Interaction of nanostructured metal overlayers with oxide surfaces. Surf. Sci. Rep..

[40] Aspromonte SG, Mizrahi MD, Schneeberger FA, Lopez JMR, Boix AV (2013). Study of the nature and location of silver in Ag-exchanged mordenite catalysts. Characterization by spectroscopic techniques. J. Phys. Chem. C.

[41] Wagner C (1975). Auger parameter in electron spectroscopy for the identification of chemical species. Anal. Chem..

[42] Ning Y (2019). A near ambient pressure photoemission electron microscope (NAP-PEEM). Ultramicroscopy.

[43] Huang W, Bao X (2001). Adsorption and reaction of CO and O_2_ on the Ag/Pt(110) surface studied by photoemission electron microscopy. Chin. Sci. Bull..

[44] Kwak JH (2009). Coordinatively unsaturated Al^3+^ centers as binding sites for active catalyst phases of platinum on γ-Al_2_O_3_. Science.

[45] Homeyer ST, Sachtler WMH (1989). Oxidative redispersion of palladium and formation of PdO particles in Nay: an application of high-precision TPR. Appl. Catal..

[46] Pettinger B, Bao X, Wilcock IC, Muhler M, Ertl G (1994). Surface-enhanced Raman scattering from surface and subsurface oxygen species at microscopically well-defined Ag surfaces. Phys. Rev. Lett..

[47] Rocha TC (2012). The silver-oxygen system in catalysis: new insights by near ambient pressure X-ray photoelectron spectroscopy. Phys. Chem. Chem. Phys..

[48] Qu Z, Huang W, Cheng M, Bao X (2005). Restructuring and redispersion of silver on SiO_2_ under oxidizing/reducing atmospheres and its activity toward CO oxidation. J. Phys. Chem. B.

[49] Zhu B, Xu Z, Wang C, Gao Y (2016). Shape evolution of metal nanoparticles in water vapor environment. Nano Lett..

[50] Zhang X (2020). Reversible loss of core–shell structure for Ni–Au bimetallic nanoparticles during CO_2_ hydrogenation. Nat. Catal..

[51] Yuan W (2020). Visualizing H_2_O molecules reacting at TiO_2_ active sites with transmission electron microscopy. Science.

[52] Fu Q (2010). Interface-confined ferrous centers for catalytic oxidation. Science.

[53] Schlögl R (2015). Heterogeneous catalysis. Angew. Chem. Int. Ed..

[54] Lamoth M (2019). Supported Ag nanoparticles and clusters for CO oxidation: size effects and influence of the silver–oxygen interactions. ACS Appl. Nano. Mater..

[55] Bocanegra MH, Matovic B (2009). Dense and near-net-shape fabrication of Si_3_N_4_ ceramics. Mater. Sci. Eng. A.

