# Supplementary material for: In situ identification of the metallic state of Ag nanoclusters in oxidative dispersion
Source: Nat Commun. 2021 Mar 3;12:1406. doi: 10.1038/s41467-021-21552-2 (PMC7930130; doi:10.1038/s41467-021-21552-2)
Supplement: Supplementary file 1 — Supplementary Information [file 41467_2021_21552_MOESM1_ESM.pdf]

Supplementary Information for

In situ identification of the metallic state of Ag clusters in oxidative dispersion

Rongtan Li[1,2,9], Xiaoyan Xu[3,4,9], Beien Zhu[5,6,9], Xiao-Yan Li[2,6], Yanxiao Ning[1], Rentao Mu[1], Pengfei Du[2,4], Mengwei Li[4], Huike Wang[7], Jiajie Liang[7], Yongsheng Chen[8], Yi Gao[5,6,*], Bing Yang[3,4,*], Qiang Fu[1,4,*], Xinhe Bao[1,4,*]

[1]State Key Laboratory of Catalysis, Dalian Institute of Chemical Physics, the Chinese Academy of Sciences, Dalian 116023, China.

[2]University of Chinese Academy of Sciences, Beijing 100049, China.

[3]CAS Key Laboratory of Science and Technology on Applied Catalysis, Dalian Institute of Chemical Physics, the Chinese Academy of Sciences, Dalian 116023, China.

[4]Dalian National Laboratory for Clean Energy, Dalian Institute of Chemical Physics, the Chinese Academy of Sciences, Dalian 116023, China.

[5]Interdisciplinary Research Center, Shanghai Advanced Research Institute, the Chinese Academy of Sciences, Shanghai 201210, China.

[6]Key Laboratory of Interfacial Physics and Technology, Shanghai Institute of Applied Physics, the Chinese Academy of Sciences, Shanghai 201800, China.

[7]School of Materials Science and Engineering, National Institute for Advanced Materials, Nankai University, Tianjin 300350, China.

[8]Key Laboratory of Functional Polymer Materials of Ministry of Education, College of Chemistry, Nankai University, Tianjin 300350, China.

[9]These authors contributed equally: Rongtan Li, Xiaoyan Xu, Beien Zhu

Email: gaoyi@zjlab.org.cn; byang@dicp.ac.cn; qfu@dicp.ac.cn; xhbao@dicp.ac.cn

Table of contents for the Supplementary Information:

Supplementary Methods……………………………………………………… #3

Supplementary discussion……………………………………………………. #5

Supplementary Figure 1-17………………………………………………….#7

Supplementary Table S1……………………………………………………… #15

Supplementary Methods

Synthesis of Ag nanowires. Ag nanowires (NWs) were synthesized by a modified polyol reduction method, the details of which had been described elsewhere.[1] In a typical synthesis, PVP (K-90) was dissolved in ethylene glycol (160 mL, 12.5 mg/mL) at 150 °C with constant stirring. Then, the sodium chloride in ethylene glycol (16 mL, 0.42 mg/mL) and silver nitrate in ethylene glycol (40 mL, 50 mg/mL) were added when the solution was cooled to room temperature. The mixture was heated in an oven at 110 °C for 12 h after vigorous magnetic stirring for 5 min to obtain AgNWs. The synthesized AgNWs were dispersed and stored in ethanol for follow-up experiments.

Catalytic testing. The catalytic testing was performed in a home-built reactor specifically designed for wafer samples. Briefly, the test sample is fixed on a sample stage in a quartz tube reactor with a minimized volume of 40 ml to enhance the response time (supplementary Fig. 13). The reactor is uniformly heated by resistive heating wires and the temperature is measured by a thermocouple attached to the quartz tube. Before reaction, inert gas was flushed for 2 h to eliminate contamination before reactants were introduced. The reaction products were monitored by an online mass spectrometer linked to the outlet of the reactor.

Computational Details:

All periodic density-functional theory (DFT) calculations were carried out using the Vienna *ab-initio* simulation package (VASP).[2-4] The spin-polarized setting is switched on. The interaction between core electrons and valance electrons is performed by the projector augmented wave (PAW) method.[5] The generalized gradient approximation in Perdew-Burke-Ernzerhof formulation (GGA-PBE) is used to describe the exchange-correlation interactions.[6,7] The cut-off energy of the plane wave is set to 400 eV. In the geometry optimizations, the position of atoms in the supercell is relaxed until the total energy difference falls below the 10^{-5} eV. The relaxation will stop if all forces convergence is smaller than 0.05 eV/ Å.

Three low-index surfaces, Ag(100), Ag(110), and Ag(111) are considered in this work, since these orientations could form the main stable surface for most synthetic material. Ag surfaces are constructed using slabs of six atomic layers for the Ag(100), Ag(110), and Ag(111) surfaces. The bottom two layers of atoms are fixed to mimic the bulk structure. The other four layers could be relaxed to simulate metallic layers. These 4×4 supercells contain 96 atoms with 15 Å vacuum spaces along the z-axis. The Brillouin zone integrations are performed on a Monkhorst−Pack $2 \times 2 \times 1$ k-point mesh.[8] The adsorption energies of oxygen atom and oxygen molecule on the Ag (100), (110), and (111) facets were carefully investigated. Several possible adsorption sites have been tested.

The adsorption energies of oxygen atom and oxygen molecule on the clusters of Ag_3 and Ag_8 were calculated. In order to eliminate the periodic effect, Ag clusters are placed in a cube with 10 Å. The k-points are set to $5\times5\times5$.[8]

To further simulate the effect of substrate, periodic 2-D (4×4) Si_3N_4(0001) slabs were used. These supercells are composed of four relaxed layers at the top and two fixed Si_3N_4 layers at the bottom with 15 Å vacuum spaces along the z-axis. Here the k-points are set to be $2 \times 2 \times 1$.[8] The Ag_3 and Ag_8 clusters were loaded on the Si_3N_4(0001) surface for optimizations. The adsorption energies of oxygen atoms and O_2 on the supported clusters were obtained after testing several adsorption configurations. The most stable models in all calculations are shown in supplementary Figs. 8-10.

The transition state was searched by using climbing image nudged elastic band (CI-NEB) method.[9] The reaction pathway of CO oxidation over the Ag_8 cluster supported on the Si_3N_4(0001) surface is presented in supplementary Figs. 15.

Supplementary Discussion:

Native oxide on Si_3N_4 substrate. A small amount of native oxides was identified on Si_3N_4 substrate, which is commonly observed due to long-term aging in the air.[10] During our quasi in situ and in situ experiments, the surface composition of Si_3N_4 substrate remains unchanged as verified by the constant *Si 2p*, *N 1s* and *O 1s* spectra in X-ray photoelectron spectroscopy (XPS) characterization, see supplementary Figs. 2, 4 and 5. We can thus eliminate the effect of surface composition during the dynamic dispersion of Ag.

Auger parameter for the identification of silver chemical state. For the quantification of Ag oxidation state, we employed Auger parameters i.e. the sums of Auger kinetic energy (KE) and photoelectron BE, because XPS core-level of Ag is unable to distinguish its oxide or metallic states. As shown in supplementary Fig. 17 (adopted from Handbook of X-ray Photoelectron Spectroscopy),[11] the Auger parameters of metallic Ag is around 726 eV, whereas the value for silver oxide is usually lower than 725 eV. The values of the Auger parameters can represent the degree of oxidation in Ag quantitatively which has been widely adopted in many other literatures.[12,13] Auger Parameters are often used to identify the initial effect and the final effect.[14,15] In the case of silver during our XPS experiments, the Auger parameter is the combination effect of chemical state (initial effect) and size (final effect). If we take the two effects separately, ~ ±2 eV and ~ ±1 eV shift of Auger parameter is observed for change of chemical state and size, respectively.[15-17] Identification of chemical state in our experiments with detailed discussion is described in the following. In quasi in situ experiments, the negative shift of Auger parameter is ascribed to the oxidation of silver, in which the change of chemical state (initial effect) is regarded as the dominant factor. The low Auger parameter is seen in other silver oxide systems.[18] In in situ experiments, the Auger parameter for Ag nanoclusters in O_2 at 700 K remains nearly unchanged as the pristine Ag/Si_3N_4 without any treatments(metallic). The BE shift indicates the decreasing size which may lead to the downshift of Auger parameter. The unchanged Auger parameter suggests that some electrons may transfer to Ag, which may explain

why silver can maintain the metallic state in O_2. The trend is similar to the results reported by Guo and co-workers.[13,17] Upon calcination in ultrahigh vacuum (UHV) at 700 K, the smaller Ag clusters aggregate to Ag nanoparticles with the size and state similar to Ag/Si_3N_4, so it is expected to observe the same Auger parameter with Ag/Si_3N_4. When cooling in O_2, the unchanged BE indicates that the size doesn't change. The downshift of Auger parameter[13,16] is ascribed to the oxidation, similar to quasi in situ experiments.

Pressure-dependent dispersion process. We noted a pressure dependence in oxidative dispersion of AgNWs. Compared with the oxidative treatment at 673 K by 1 mbar O_2, the higher Ag/Si ratio by the treatment in 1 bar O_2 indicates the stronger dispersion (supplementary Fig. 14). It should be ascribed to the stronger driving force due to more oxygen adsorption. Though XPS experiment for 1 bar pressure is still technically limited by the current NAP-XPS technique, the previous works have confirmed that increasing the O_2 pressure from 1 mbar to 1 bar at 673 K will lead to higher coverage of adsorbed oxygen but not the oxidation[19] and Ag_2O decomposes at the temperatures of 673 K in air.[20] Therefore, from the thermodynamic point of view the same mechanism should be valid in 1 bar O_2 as that in 1 mbar O_2.

Reproducibility and durability. The reproducibility and durability of Ag/Si_3N_4 catalyst have been tested under CO oxidation reaction conditions. As presented in Fig. 5 and supplementary Fig. 11, the similar hysteresis phenomenon was observed in a second Ag/Si_3N_4 catalyst, showing an identical dispersion phenomenon with good reproducibility. The time-on-stream stability test of Ag/Si_3N_4 was further performed at 673 K. The Ag/Si_3N_4 catalyst showed excellent durability under the reaction condition up to 35 hr with a small decrease by 4%. The results showed that the O_2-induced dispersion led to increasing activity and high stability of Ag/Si_3N_4 catalysts in O_2-rich reaction atmosphere.

Effect of Ag density. The effect of surface concentration of Ag is further investigated

as an important factor during dispersion process. As shown by XPS (Fig. 1e, Fig. 3b and Fig. 4c.) and SEM (Fig. 1c and supplementary Fig. 16) results, the three samples with low, medium and high Ag densities showed the same trend of behavior/dynamics during dispersion, despite only slight difference in the dispersion rate (duration to reach equilibrium) and dispersion degree. All samples were dispersed into small clusters/particles after the O_2 treatment and become invisible under SEM imaging. We can thus safely conclude that the surface concentration has minor effect on the dispersion dynamics under our experimental conditions.

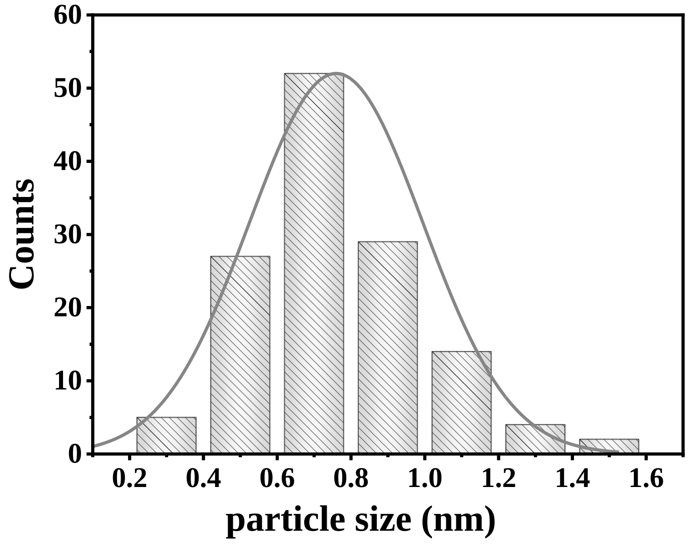

Supplementary Figure 1. Particle size distribution of highly dispersed Ag nanoclusters after oxidative dispersion. Statistics of HAADF-STEM images show a mean particle size of 0.7 nm, with a significant number of sub-nm Ag_n clusters and single atoms.

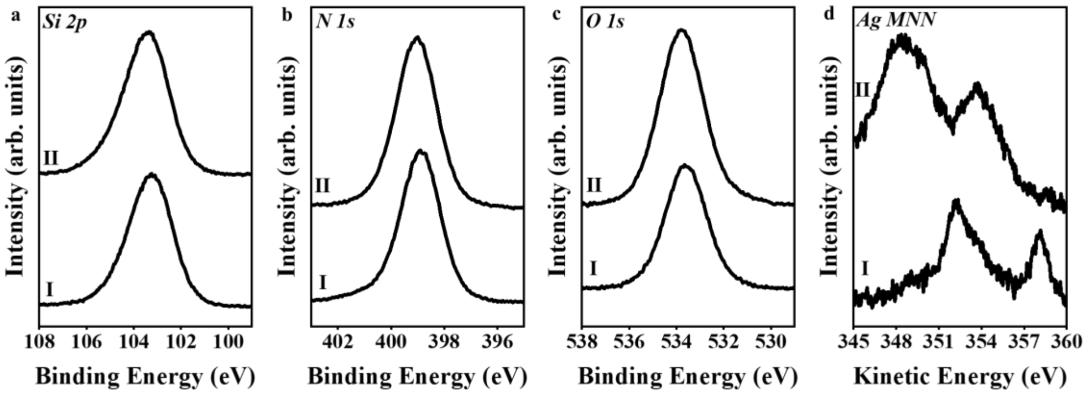

Supplementary Figure 2. XPS spectra of AgNWs before and after oxidative dispersion. a *Si 2p*, **b** *N 1s*, **c** *O 1s* and **d** *Ag MNN* Auger spectra of pristine Ag/ Si_3N_4 (I) and Ag/Si_3N_4 -O-673 (II) samples. The identical XPS spectra suggest the stability of Si_3N_4 substrate during the oxidation treatment. *O1s* signal is from the native surface oxide of the Si_3N_4 film. The *Ag MNN* Auger spectra of sample I is acquired from the Ag/Si_3N_4 with high Ag density (see method for detail). See supplementary discussion for detailed analysis of surface composition of Si_3N_4 substrate.

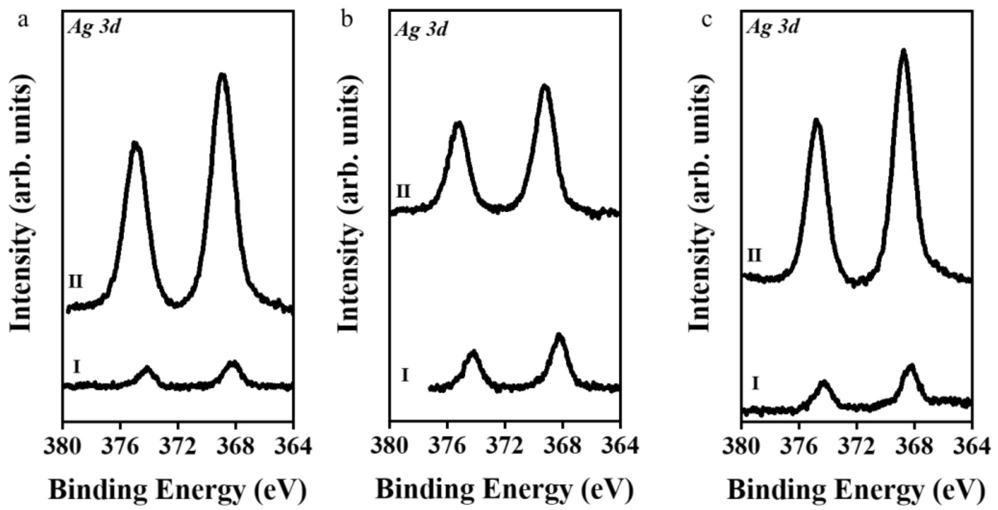

Supplementary Figure 3. Dispersion behavior of Ag on different supports. XPS *Ag 3d* spectra of Ag/oxide surfaces before and after the oxygen treatment in O_2 at 673 K: **a** SiO_2/Si(111); **b** Al_2O_3(0001) and **c** $SrTiO_3$(110). The bottom spectra and upper spectra are the *Ag 3d* spectra of Ag/oxide surfaces before (I) and after the oxygen treatment (II) respectively.

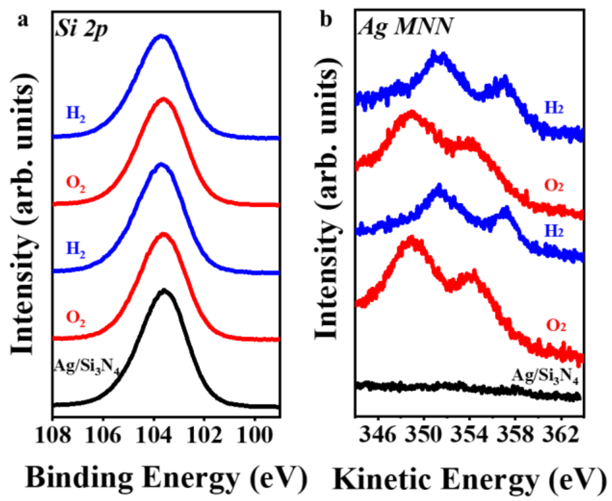

Supplementary Figure 4. XPS spectra of Ag/Si_3N_4 during redox cycles. **a** *Si 2p* and **b** *Ag MNN* Auger spectra of Ag/ Si_3N_4 upon cyclic redox treatments.

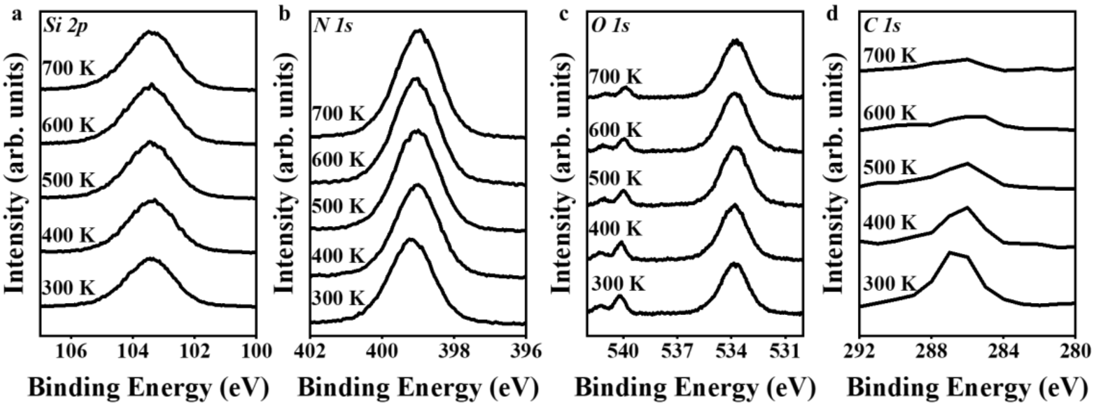

Supplementary Figure 5. XPS spectra of Ag/Si_3N_4 during *in situ* oxidative dispersion in 1 mbar O_2. **(a)** *Si 2p,* **(b)** *N 1s,* **(c)** *O 1s,* and **(d)** *C 1s* XPS spectra of Ag/Si_3N_4 upon heating in 1 mbar O_2 from 300 to 700 K.

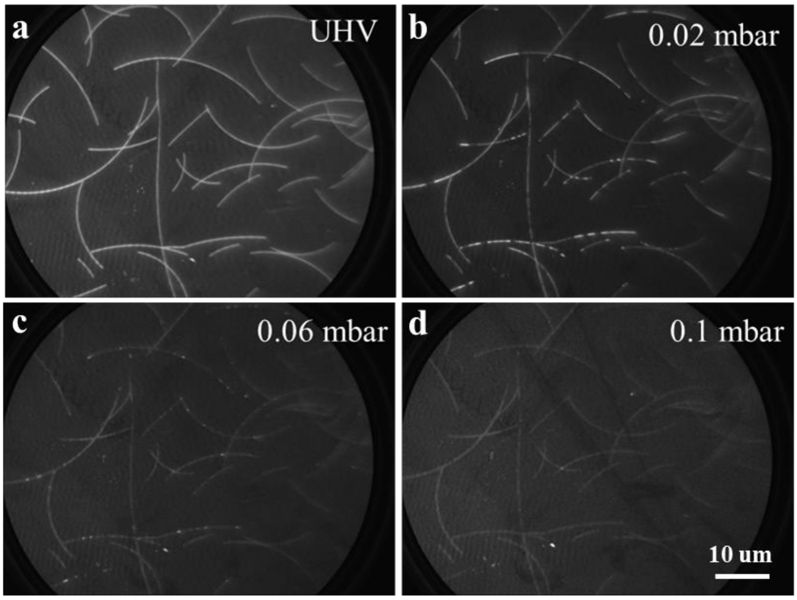

Supplementary Figure 6. NAP-PEEM images of Ag/Si_3N_4 during heating in O_2 with increasing O_2 pressure at 900 K. a UHV; **b** 0.02 mbar O_2; **c** 0.06 mbar O_2; **d** 0.1 mbar O_2. FOV = 70 µm. The experiment was conducted by dosing increasing amount of O_2 until 0.1 mbar at the specific temperature and the detail can be seen in method.

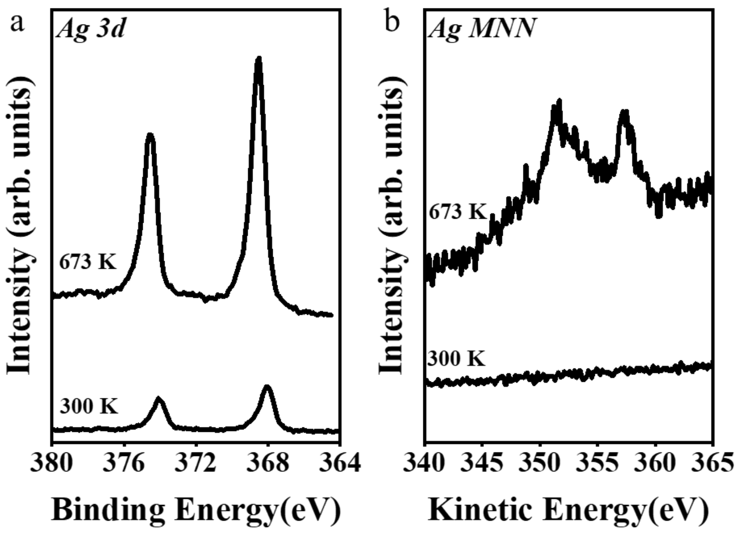

Supplementary Figure 7. In situ XPS study of dispersion behavior of Ag on SiO_2 support. a *Ag 3d* XPS spectra and **b** *Ag MNN* Auger spectra of Ag-SiO_2 annealing in 1 mbar O_2.

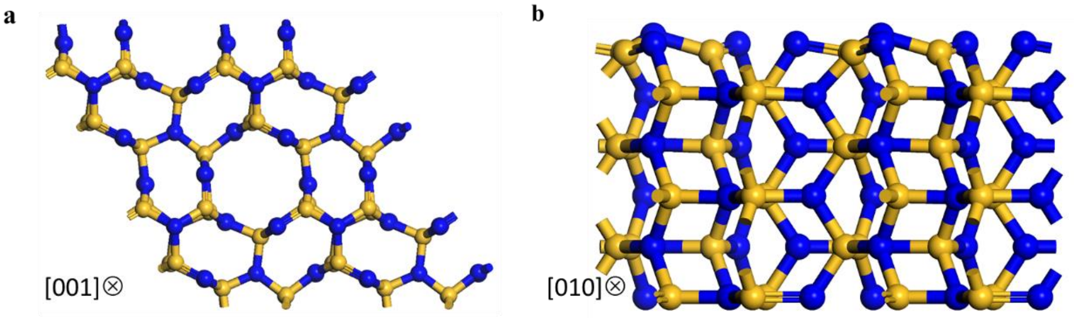

Supplementary Figure 8. The atomic structure of Si_3N_4(0001) slab. a The top view; **b** The side view. Yellow: Si; blue: N.

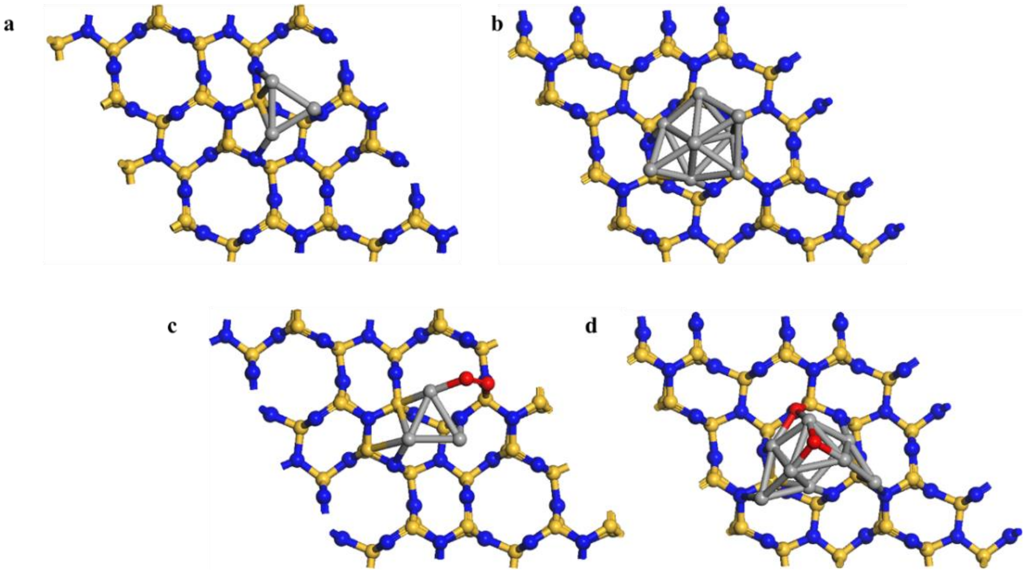

Supplementary Figure 9. The atomic structures of Ag_3 cluster and Ag_8 cluster under vacuum and O_2 environment. **a** and **b** are Ag_3 and Ag_8 loaded on the $Si_3N_4(0001)$ surface. **c** and **d** show that Ag clusters adsorb a molecular oxygen and two oxygen atoms with the largest adsorption energies, respectively. Red: O; grey: Ag; yellow: Si; blue: N.

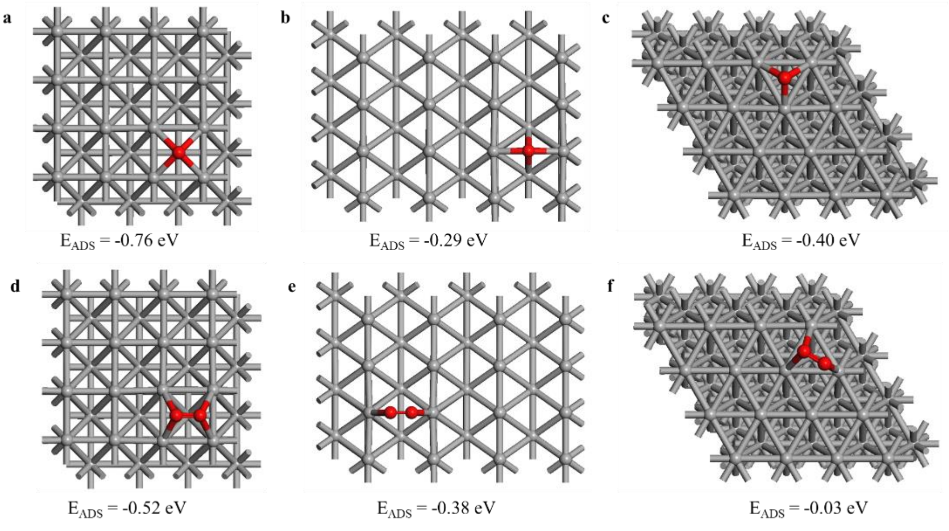

Supplementary Figure 10. The most stable configurations of oxygen atom or molecule adsorbed on three low-index Ag surfaces. **a** O atom-Ag(100); **b** O atom-Ag(110); **c** O atom-Ag(111); **d** O_2-Ag(100); **e** O_2-Ag(110); **f** O_2-Ag(111). Red: O; grey: Ag.

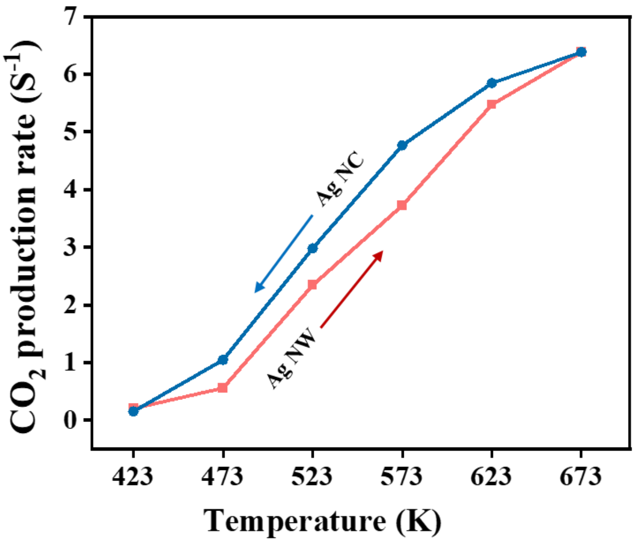

Supplementary Figure 11. Reproducibility of Ag/Si_3N_4 catalysts. The repeatability of CO_2 productivity of Ag/Si_3N_4 and Ag nanoclusters in CO oxidation reaction.

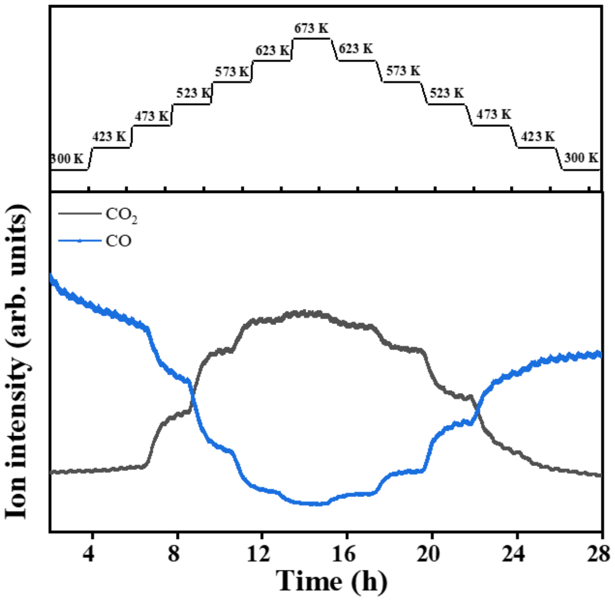

Supplementary Figure 12. Catalytic performance of Ag/Si_3N_4 during CO oxidation reaction. Real-time ion intensity of CO and CO_2 during reaction monitored by online mass spectrometer and the temperature ramp of stepwise heating/cooling cycles.

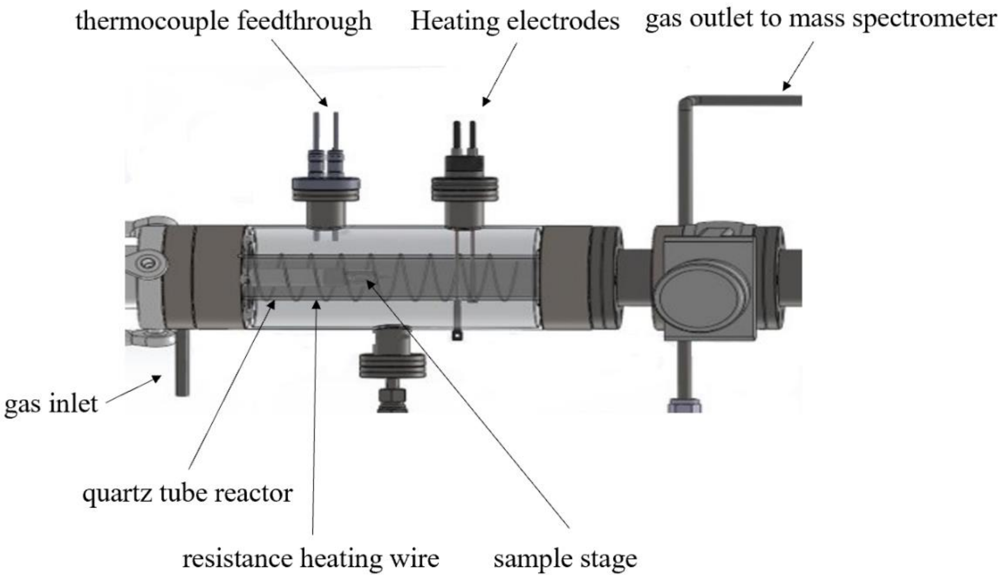

Supplementary Figure 13. Scheme of the home-built reactor. See details of the reactor in supplementary methods.

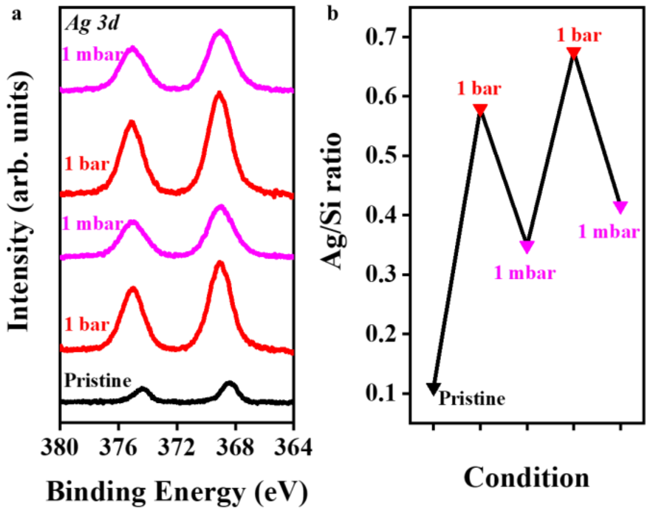

Supplementary Figure 14. ***Ag 3d*** **XPS spectra of Ag/ Si_3N_4 upon oxidation in 1 bar and 1 mbar O_2 at 673 K. a** *Ag 3d* XPS spectra and **b** Ag/Si ratio. The intensity is normalized by *Si 2p* peak intensity and further reflects the higher Ag dispersion in 1 bar O_2.

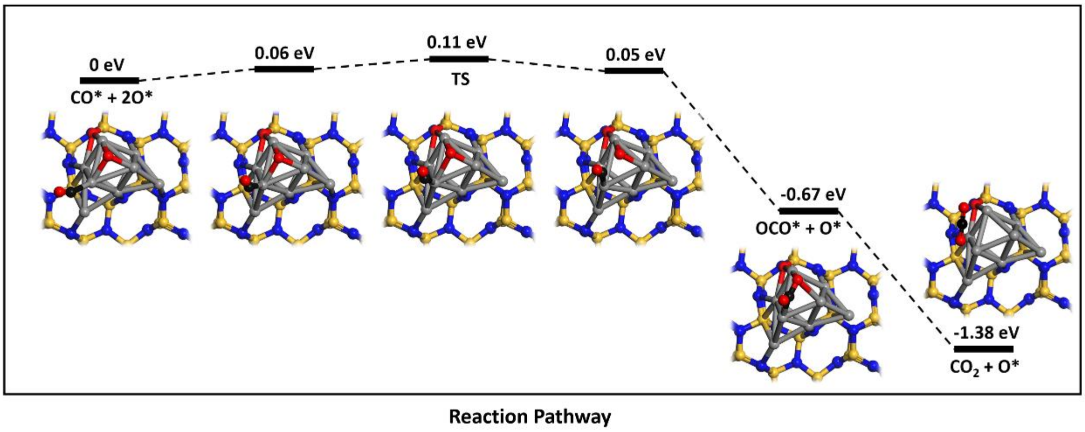

Supplementary Figure 15. The reaction pathway for CO oxidation on the Ag8 cluster is presented. The energy given in figure is the energy relative to CO*+O*. Red: O; grey: Ag; yellow: Si; blue: N, black, C.

(1) $O_2(g) \leftrightarrow 2O^*$

(2) $CO(g) \leftrightarrow CO^*$

(3) $O^* + CO^* \leftrightarrow OCO^*$ $\quad K_1$

(4) $OCO^* \leftrightarrow CO_2(g) + *$

The reaction rate constant K_1 can be calculated based on transition state theory.

$$K_1 = \frac{k_b T}{h}\frac{q_{TS}}{q_{CO*}q_{O*}}\exp(\frac{-\Delta E_{TS}}{k_b T})$$

q_i is the partition function of difference species, including rotational, transitional and vibrational part. Here, it was assumed $\frac{q_{TS}}{q_{CO}q_O} \approx 1$. Finally, we can obtain

$$K_1 = 2.10 \times 10^{12}/\text{s} \quad (\Delta E_{TS} = 0.11\ eV, T = 673\ K)$$

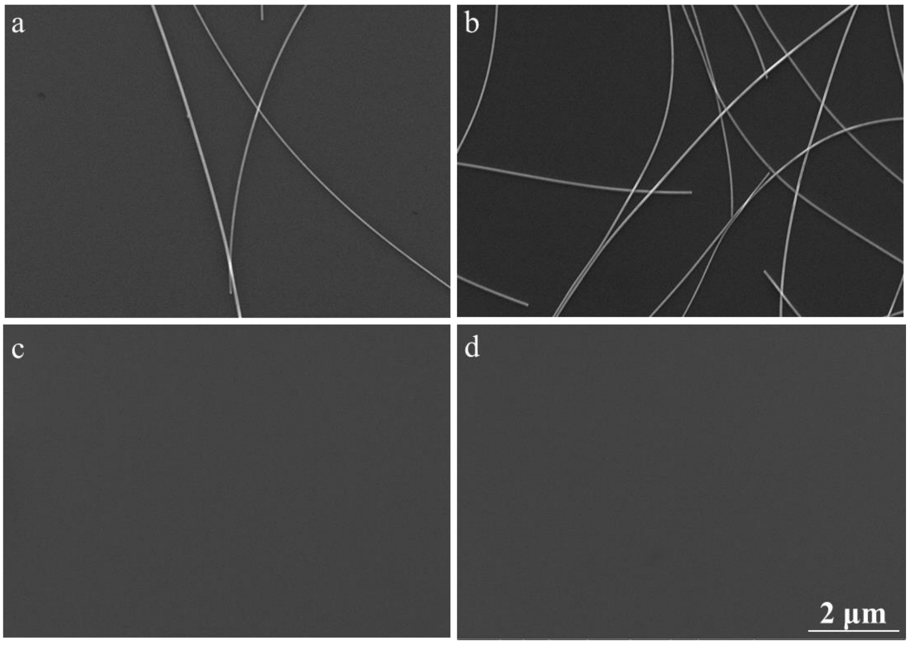

Supplementary Figure 16. a and **c** SEM images of medium density Ag/Si_3N_4 and Ag/Si_3N_4-O-673. **b** and **d** SEM images of high density Ag/Si_3N_4 and Ag/Si_3N_4-O-673. All scale bar is 2 um.

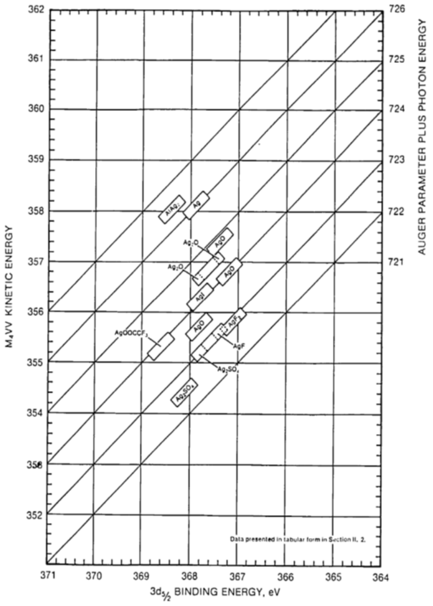

Supplementary Figure 17. Wagner plot for Ag. (The whole image is adapted with permission from ref[11] from John Wiley and Sons)

Supplementary Table 1. XPS results of Ag/SiO_2 annealing in 1 mbar O_2.

Temperature	$Ag\ 3d_{5/2}$/eV	$Ag\ M_4N_5N_5$/eV	Auger parameter/eV
300 K	368	---	726 (previous results)
673 K	368.5	357.2	725.7

References

1 Wang, H., Tang, H., Liang, J. & Chen, Y. Dynamic agitation-induced centrifugal purification of nanowires enabling transparent electrodes with 99.2% transmittance. *Adv. Funct. Mater.* **28**, 1804479 (2018).

2 Kresse, G., Hafner, J. Ab initio molecular dynamics for liquid metals. *Phys. Rev. B* **47**, 558-561 (1993).

3 Kresse, G., Hafner, J. Ab initio molecular-dynamics simulation of the liquid-metal-amorphous-semiconductor transition in germanium. *Phys. Rev. B* **49**, 14251-14269 (1994).

4 Kresse, G., Fuethmiiller, J. Efficiency of ab-initio total energy calculations for metals and semiconductors using a plane-wave basis set. *Comp. Mater. Sci.* **6**, 15-50 (1996).

5 Kresse, G., Joubert, D. From ultrasoft pseudopotentials to the projector augmented-wave method. *Phys. Rev. B* **59**, 1758-1775 (1999).

6 Perdew, J. P., Burke, K., Ernzerhof, M. Generalized gradient approximation made simple. *Phys. Rev. Lett.* **77**, 3865-3868 (1996).

7 Blochl, P. E., Jepsen, O. & Andersen, O. K. Improved tetrahedron method for Brillouin-zone integrations. *Phys. Rev. B* **49**, 16223-16233 (1994).

8 Monkhorst, H. J. & Pack, J. D. Special points for Brillouin-zone integrations. *Phys. Rev. B* **13**, 5188-5192 (1976).

9 Henkelman, G.; Uberuaga, B. P. & Jónsson, H. A. Climbing image nudged elastic band method for finding saddle points and minimum energy paths. *J. Chem. Phys.* ***113***, 9901(2000).

10 Raider, S. I., Flitsch, R., Aboaf, J. A. & Pliskin, W. A. Surface oxidation of silicon nitride films. *J. Electrochem. Soc.* **123**, 560-565 (1976).

11 Briggs, D. Handbook of X-ray photoelectron spectroscopy, ed. C. D. Wanger, W. M. Riggs, L. E. Davis, J. F. Moulder and G. E. Muilenberg Perkin-Elmer Corp., Physical Electronics Division, Eden Prairie, Minnesota, USA, 1979. **3**, 1-190 (1981).

12 Aspromonte, S. G., Mizrahi, M. D., Schneeberger, F. A., Lopez, J. M. R. & Boix, A. V. Study of the nature and location of silver in Ag-exchanged mordenite catalysts. Characterization by spectroscopic techniques. *J. Phys. Chem. C* **117**, 25433-25442 (2013).

13 Huang, Z. *et al.* Catalytically active single-atom sites fabricated from silver particles. *Angew. Chem. Int. Ed.* **51**, 4198-4203 (2012).

14 Moretti, G. The Wagner plot and the Auger parameter as tools to separate initial- and final-state contributions in X-ray photoemission spectroscopy. *Surf. Sci.* **618**, 3-11 (2013).

15 Luo, K. *et al.* The growth of silver on an ordered alumina surface. *J. Phys. Chem. B* **109**, 4064-4068 (2005).

16 Schon, G. ESCA studies of Ag, Ag_2O and AgO. *Acta. Chem. Scand.* **27**, 2623-2633 (1973).

17 Guo, D., Guo, Q., Zheng, K., Wang, E. & Bao, X. Initial growth and oxygen adsorption of silver on Al_2O_3 film. *J. Phys. Chem. C* **111**, 3981-3985 (2007).

18 Hu, P. *et al.* Electronic metal-support interactions in single-atom catalysts. *Angew. Chem. Int. Ed.* **53**, 3418-3421 (2014).

19 Li, W., Stampfl, C. & Scheffler, M. Why is a noble metal catalytically active? The role of the O-Ag interaction in the function of silver as an oxidation catalyst. *Phys. Rev. Lett.* **90**, 256102 (2003).

20 Waterhouse, G. I. N., Bowmaker, G. A. & Metson, J. B. The thermal decomposition of silver (I, III) oxide: A combined XRD, FT-IR and Raman spectroscopic study. *Phys. Chem. Chem. Phys.*

3, 3838-3845 (2001).
