# Supplementary material for: In situ identification of the metallic state of Ag nanoclusters in oxidative dispersion
Source: Nat Commun. 2021 Mar 3;12:1406. doi: 10.1038/s41467-021-21552-2 (PMC7930130; doi:10.1038/s41467-021-21552-2)
Supplement: Supplementary file 3 — Description of Additional Supplementary Files [file 41467_2021_21552_MOESM3_ESM.pdf]

Description of Additional Supplementary Files

File Name: Supplementary Movie 1
Description: ESEM imaging of a AgNWs/Si_3N_4 surface annealed in 1 mbar O_2 at 673 K.
