# Supplementary material for: In situ identification of the metallic state of Ag nanoclusters in oxidative dispersion
Source: Nat Commun. 2021 Mar 3;12:1406. doi: 10.1038/s41467-021-21552-2 (PMC7930130; doi:10.1038/s41467-021-21552-2)
Supplement: Supplementary file 2 — Peer Review File [file 41467_2021_21552_MOESM2_ESM.pdf]

Reviewer #1 (Remarks to the Author):

This work reported an in situ characterizations of the oxidative dispersion of Ag nanostructures on Si_3N_4 surface during CO oxiation. A typical oxidative dispersion process Ag remains in the metallic state due to the chemisorption of O_2. It is interesting to see that the Ag nanoclusters could be oxidized upon cooling in the O_2 atmosphere. The mechanism was explained by dynamic equilibrium considering temperature and pressure and the authors proposed that such mechanism could be employed to further demonstrate the oxidative dispersion of many other metals. The authors claimed the importance of the new data. Specific comments are listed below.
(1) A brief description of the synthesis process of Ag nanowires should be given in SM.
(2) What about the desorption energies and reaction rates?
(3) How to consider the surface defects as the defects may affect the reaction mechanism dramatically?
(4) Although the authors tried explaining the reaction mechanism, the reactivity of CO_2 production is not so attractive compared to other catalysts. Also, the durability and reproducibility of the obtained results were not provided.

Reviewer #2 (Remarks to the Author):

In the manuscript "In situ identification of the metallic state of Ag nanoclusters in oxidative dispersion" the Dalian groups with correspondign authours Gao, Yang, Fu and Bao present an interesting work on the dispersion of silver-nanowires on a Si_3N_4-support under oxidizing conditions. Such sintering and (re)-dispersion studies are extremely important in catalysis and need further attention to obtain stable catalysts. The Dalian groups are well known in the field of single site catalysis, high-end characterization and heterogeneous catalysis in general. For this study, state of the art and highly attractive methods like NAP-XPS and ESEM were used to in situ follow the dispersion process of Ag. As model reaction CO-oxidation was used. The main outcome of the study is that the dispersion of silver is observed under oxidizing conditions whereas aggregation of the Ag nanostructures is observed under more reducing conditions.

I have the following comments:

1) This dispersion/redispersion is a concept that has been reported in literature as also outlined by the authors, e.g. the intelligent catalysts by Toyota (ref. 8). It has also been the topic of several review articles (e.g. ref. 3 and 4). In addition to the studies given in the manuscript, also a few further may be cited (e.g. L. Kibis, J. Phys Chem. C, 10.1021/acs.jpcc.7b09983) The authors claim that the understanding has been "established mainly based on ex situ spectroscopic characterization…" (first page of "main") and in the abstract they write "Lacking in situ characterizations, however, the interplay …" has not been well illustrated.
I disagree with these statements, as there are various studies that report "in situ" studies, e.g. Yasutaka Nagai et al. report in Ang. Chem. (doi.org/10.1002/anie.200803126, 2008) oxidative redispersion of Pt nanoparticles supported on ceria-based oxide in situ XANES analysis. An atomic migration model accounts for the observed redispersion through the trapping of Pt species at sites on the Ce support that exhibit strong interactions between the Pt oxide and the support. There are various further in situ studies like in situ TEM (ref. 9) that show redispersion in oxygen and aggregation in reducing conditions, which was further extended e.g. by G. Ferre et al. recently (Catal. Sci Technol. , doi: 10.1039/d0cy00732c). Also, on Pt/TiO_2 (L. DeRita, et al., Nature Materials volume 18, pages746–751(2019)) in situ studies have been reported. A good additional study is on Rh-based ceria catalysts by Ikemoto et al., also studying the reversible behavior by in situ spectroscopies (PCCP, 2019, doi.org/10.1039/C9CP04625A).
Turning to silver-based catalysts as outlined in this study, also in this case the dynamics has been reported before, also by in situ TEM (e.g., Y. Gao, Catal. Sci. Technol., 10.1039/c7cy00831g, 2017, soot oxidation e.g. D. Gardini et al., Appl. Catal. B, 10.1016/j.apcatb.2015.10.029, 2016). Hence, the novelty of the manuscript needs to be sharpened and it is therefore not acceptable in its present form.

2) Also other groups like the Schlögl group have been very active in the area of silver catalysed oxidation and need attention. Maybe I overlooked a corresponding reference as mostly the first authour is only given.
3) The dispersion of Ag and its dispersion is described to occur on reduced Ag species with "adsorbed" oxygen rather than oxidized Ag. However, this claim is not clear for me and also not sufficiently convincing for me, as no quantification of the oxidation state of the Ag-species are performed with XPS (only the position of the peak maximum is presented). Note also that in the abstract the authors write "…we unravel that in such a typical oxidative dispersion process Ag actually remains in its metallic state and the dispersion is caused by the chemisorption of O2." A chemisorption – so the formation of a chemical bond - of O2 is an oxidation if considering cluster/particles. The authors should overthink their wording. They state that Ag is not oxidized, but it is still called a "oxidative dispersion". This is contradictory.
4) When reading the manuscript, it becomes not clear that 3 different type of samples were used for the characterization and not only 1 sample. Especially for deducting mechanistic insights, this is to my opinion misleading, since due to the different surface concentrations, the samples can behave as well differently. In the experimental part it is written: "prepared three kind of samples with different Ag density (high, medium, and low). To acquire strong Auger spectra, samples with high NW density were used and shown in Fig. 4. Samples with medium AgNW density were used to get obvious change of Ag/Si ratio during various treatments, also to make XPS peak intensity strong enough, shown in Figs. 1-3."
5) Comment to the conclusion: "We provide a solid evidence for the fact that oxide formation is not necessary in oxidative dispersion, but a result during cooling in O2." This is contradictory to the DFT results, where it was stated that "On the contrary, associative O2 and dissociative O atoms adsorb stably at the Ag3-Si3N4 ($Eads\ clu$ = -2.24 eV) and Ag8-Si3N4 ($Eads\ clu$ = -3.97 eV) interfaces". The provided data does suggest, that a surface oxidation (nothing else is a O2 chemisorption of "dissociative O atoms") is needed for the dispersion and not a bulk oxidation. As mentioned before, the wording has to be improved and the terminology should be defined to enhance the clearness of the manuscript.

In summary, the manuscript is not recommended for publication in Nature Communications in its present version because the novelty is not clear enough yet, the process of redispersion (chemisorption / oxidation) and also some experimental details/choice of the experimental study (systematics) are not clear.

Reviewer #3 (Remarks to the Author):

It is an interesting and solid piece of study where in situ XPS technique has been applied to study the "oxidative dispersing" of Ag particles. It was observed that metallic Ag, instead of Ag oxide, was the main species being dispersed. The authors further confirmed that the chemisorption of O2 is the driving force for the re-dispersion of metallic Ag clusters. While the study is thorough, and self-consistent, its wide applicability and general validity remains a question. What is reported here may represent a special case rather than a general phenomenon.

First of all, Si3N4 is not a typical support. Ag/Si3N4 is not a widely used commercial catalyst. Ag/Si3N4 is not a representative system. There is no guarantee that the dispersion behaviour of metal on non-oxides to be the same as on oxides. Further, oxidative re-dispersion is normally conducted at 1 bar in air. The concentration of O2 is orders of magnitude higher than the O2 used in in situ XPS. This will change hugely the oxidation behaviour of Ag. Unless the same observation is obtained on a more typical metal oxide support under conditions closer to general practise, the implication of the study is compromised. Consider doing this requires effort beyond a major revision, I would suggest the authors to go for a more specialized journal. It is a very well conducted piece, and I feel it can be published in any surface chemistry journals with essentially no revision.

Reviewer #1:

This work reported an in situ characterizations of the oxidative dispersion of Ag nanostructures on Si_3N_4 surface during CO oxidation. A typical oxidative dispersion process Ag remains in the metallic state due to the chemisorption of O_2. It is interesting to see that the Ag nanoclusters could be oxidized upon cooling in the O_2 atmosphere. The mechanism was explained by dynamic equilibrium considering temperature and pressure and the authors proposed that such mechanism could be employed to further demonstrate the oxidative dispersion of many other metals. The authors claimed the importance of the new data.

Response: We thank the referee for the positive evaluation of our work.

Specific comments are listed below.

1) A brief description of the synthesis process of Ag nanowires should be given in SM.

Response: We thank the referee for the suggestion. A brief description of the synthesis process of Ag nanowires has been added in the revised SI on **Page 3**. Also see as below: "In a typical synthesis, PVP (K-90) was dissolved in ethylene glycol (160 mL, 12.5 mg /mL) at 150 °C with constant stirring. Then, the sodium chloride in ethylene glycol (16 mL, 0.42 mg/mL) and silver nitrate in ethylene glycol (40 mL, 50 mg/mL) were added when the solution was cooled to room temperature. The mixture was heated in an oven at 110 °C for 12 h after vigorous magnetic stirring for 5 min, to obtain AgNWs."

2) What about the desorption energies and reaction rates?

Response: We thank the referee for this question. We have carried out additional DFT calculations for the desorption energy and reaction rate using the model of Ag_8 cluster. The energy pathway of the reaction is shown in Fig. R1. The energy barrier during the reaction is 0.11 eV, in the elementary step of forming adsorbed OCO* from adsorbed O* and CO*. According to the transition state theory, the reaction rate constant of this step is calculated as $2.10 \times 10^{12}/\mathrm{s}$ at 673 K.

The details for this calculation are shown below and added to the revised SI on **Page 14** (supplementary Fig. 16). We also found that the formed CO_2 is readily to desorb from the cluster. The energy of the system becomes lower after the desorption.

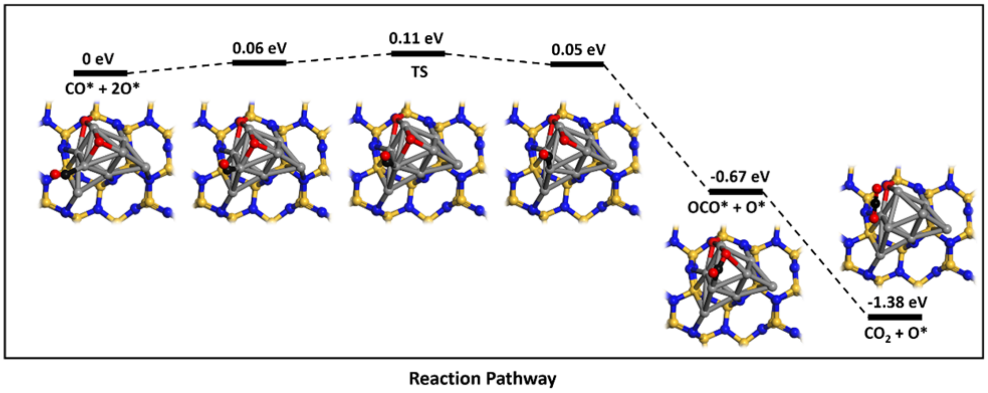

Figure R1. The reaction pathway for CO oxidation on the Ag_8 cluster. The energy values given in the figure are relative to CO* + O*. Red: O; grey: Ag; yellow: Si; blue: N; black: C.①

O_2 (g) + 2* → 2O*; ② CO (g) + * → CO*; ③ O* + CO* → OCO*, K_1; ④OCO* → CO_2 (g) + *.

The reaction rate constant K_1 can be calculated based on the transition state theory:

$$K_1 = \frac{k_b T}{h} \frac{q_{TS}}{q_{CO*} q_{O*}} \exp(\frac{-\Delta E_{TS}}{k_b T}).$$

q_i is the partition function of the difference species, including rotational, transitional and vibrational parts. Here, it was assumed $\frac{q_{TS}}{q_{CO} q_O} \approx 1$. Finally, we can obtain

$$K_1 = 2.10 \times 10^{12}/\mathrm{s} \quad (\Delta E_{TS} = 0.11\ eV, T = 673\ K)$$

3) How to consider the surface defects as the defects may affect the reaction mechanism dramatically?

Response: We fully agree with the referee on this point that surface defects may dramatically affect the reaction mechanism, particularly on reducible oxides. However, Si_3N_4 is a very inert material containing rare surface defects and can remain stable under harsh conditions [Ref. 55]. This is also confirmed by our XPS results (see Fig.

R2 and supplementary Fig. 4) that the chemical state (Si 2p and N 1s) of Si_3N_4 surface remains unchanged in O_2 up to 700 K. Considering the inert nature of Si_3N_4 surface, we believe that the surface defects may have very limited effect on both dispersion and reactivity of the Ag/Si_3N_4 catalyst.

To further emphasize this point, we have modified the text as "The inert Si_3N_4 surfaces are nearly free of surface defects and remain stable under the reaction conditions (see supplementary discussion)." in the revised manuscript on **Page 15**.

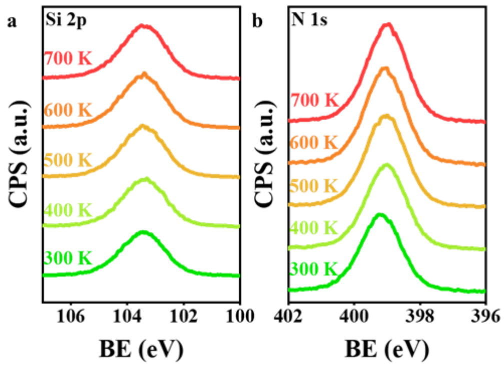

Figure R2. XPS spectra of Ag/Si_3N_4 surface during *in situ* oxidative dispersion. (a) Si 2p and (b) N 1s XPS spectra of the Ag/Si_3N_4 surface upon heating in 1 mbar O_2 from 300 to 700 K.

4) Although the authors tried explaining the reaction mechanism, the reactivity of CO_2 production is not so attractive compared to other catalysts.

Response: We thank the referee for pointing this out. We are aware that Ag is not usually considered as an excellent metal catalyst for low temperature CO oxidation. In case of our Ag/Si_3N_4 model catalyst of this work, the low temperature activity (CO conversion) is not so efficient largely due to the ultra-small metal loading of only 0.001mg per sample. (see Fig. R3a) Considering the space velocity and ultra-small metal loading, our Ag/Si_3N_4 model catalyst with highly dispersed ultra-small Ag clusters actually presents a decent COOX activity. To further demonstrate this point, we have successfully transferred this concept to a real powder catalyst by preparing 5%wt. Ag catalyst supported on SiO_2 material with high surface area. The similar dispersion behavior of Ag has been observed after high temperature treatment in

O_2-rich reaction atmosphere, and the highly dispersed Ag/SiO_2 catalyst exhibits a remarkably activity with 100% CO conversion at 343 K (Fig. R3b) which is actually comparable to other reported catalysts (*Appl. Catal. A: Gen.* 2019, 570, 51–61; *Catal. Tod.* 2019, 332, 189–194).

To address this point, we have replaced the CO_2 production rate with CO conversion in Fig. 5b and the corresponding description in the revised manuscript on **Page 13** as "The highly dispersed Ag nanoclusters formed during reaction exhibit an enhanced activity especially at low temperature, higher than the initial state of AgNWs upon heating." We have also modified the supplementary Fig. 12 to show mass spectroscopy results of both CO and CO_2 in the revised SI on **Page 12.**

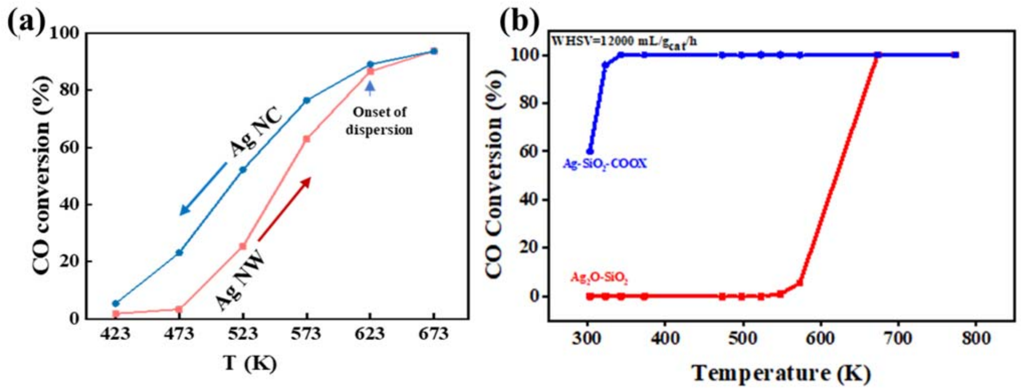

Figure R3. Catalytic COOX performance. (a) The CO conversion for Ag/Si_3N_4 upon the heating and cooling cycle; **(b)**The CO conversion over an Ag/SiO_2 powder catalyst and the catalyst after dispersion treatment in O_2-rich reaction atmosphere (Ag/SiO_2-COOX). The Ag-SiO_2-COOX catalyst shows a remarkable activity with 100% conversion at 343 K, which is significantly lower than that of the Ag/SiO_2 catalyst at around 400 K. The Ag_2O-SiO_2 is the mixture of commercial Ag_2O and SiO_2 powders. The AgO-SiO_2-COOX is obtained from the Ag_2O-SiO_2 catalysts after the COOX reactions up to 773 K. (Confidential for review purpose only)

Also, the durability and reproducibility of the obtained results were not provided.

Response: To address the durability and reproducibility of our results, we have repeated the activity measurements and performed a time-on-stream stability test of

the Ag/Si_3N_4 sample at 673 K. As presented in Fig. R4, our Ag/Si_3N_4 catalyst shows very good reproducibility and excellent durability under reaction condition up to 35 hr with only mild decline by 4%.

To address this point, we have added a new figure 5c and revised the manuscript accordingly on **Page 13** as "The time-on-stream stability test in Fig. 5c further reveals an excellent durability of highly dispersed Ag/Si_3N_4 catalyst under the reaction condition (at 673 K) up to 35 hr (also see supplementary discussions). Our results thus prove the long-term stability of dispersed nearly-metallic Ag atoms/clusters under O_2-rich reaction environment, and open up a new route for the fabrication of highly/atomically dispersed catalyst during reaction."

In addition, we added a new figure of supplementary Fig. 11 (Fig. R4) in the revised SI on **Page 12**, as well as an additional discussion to address the reproducibility and durability in the revised SI on **Page 6**.

"**Durability and reproducibility and durability.** The reproducibility and durability of Ag/Si_3N_4 catalysts have been tested under CO oxidation reaction conditions. As presented in supplementary Fig. 1, the similar hysteresis phenomenon was observed in a second Ag/Si_3N_4 catalyst, showing an identical dispersion phenomenon with good reproducibility. The time-on-stream stability test of Ag/Si_3N_4 was further performed at 673 K. The Ag/Si_3N_4 catalyst showed excellent durability under the reaction condition up to 35 hr with a small decrease by 4%. The results showed that the O_2-induced dispersion led to increasing activity and high stability of Ag/Si_3N_4 catalysts in O_2-rich reaction atmosphere."

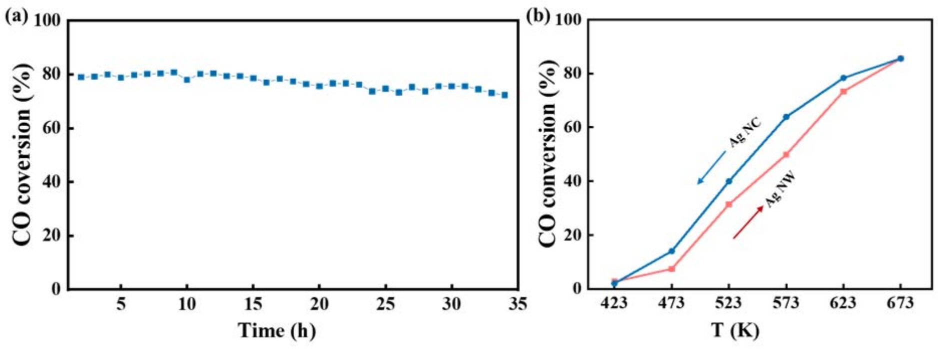

Figure R4. Durability and reproducibility of Ag/Si_3N_4 catalysts. (a) The time-on-stream stability test of Ag/Si_3N_4 catalyst under reaction condition at 673 K. **(b)** The catalytic activity of a second Ag/Si_3N_4 catalyst for CO oxidation reaction with good reproducibility.

Reviewer #2:

In the manuscript "In situ identification of the metallic state of Ag nanoclusters in oxidative dispersion" the Dalian groups with corresponding authors Gao, Yang, Fu and Bao present an interesting work on the dispersion of silver-nanowires on a Si_3N_4-support under oxidizing conditions. Such sintering and (re)-dispersion studies are extremely important in catalysis and need further attention to obtain stable catalysts. The Dalian groups are well known in the field of single site catalysis, high-end characterization and heterogeneous catalysis in general. For this study, state of the art and highly attractive methods like NAP-XPS and ESEM were used to in situ follow the dispersion process of Ag. As model reaction CO-oxidation was used. The main outcome of the study is that the dispersion of silver is observed under oxidizing conditions whereas aggregation of the Ag nanostructures is observed under more reducing conditions.

Response: We thank the referee for the positive evaluation of our work.

I have the following comments:

1) This dispersion/redispersion is a concept that has been reported in literature as also outlined by the authors, e.g. the intelligent catalysts by Toyota (ref. 8). It has also been the topic of several review articles (e.g. ref. 3 and 4). In addition to the studies given in the manuscript, also a few further may be cited (e.g. L. Kibis, J. Phys Chem. C, 10.1021/acs.jpcc.7b09983) The authors claim that the understanding has been "established mainly based on ex situ spectroscopic characterization…" (first page of "main") and in the abstract they write "Lacking in situ characterizations, however, the

interplay …" has not been well illustrated.

I disagree with these statements, as there are various studies that report "in situ" studies, e.g. Yasutaka Nagai et al. report in Ang. Chem. (doi.org/10.1002/anie.200803126, 2008) oxidative redispersion of Pt nanoparticles supported on ceria□based oxide in situ XANES analysis. An atomic migration model accounts for the observed redispersion through the trapping of Pt species at sites on the Ce support that exhibit strong interactions between the Pt oxide and the support. There are various further in situ studies like in situ TEM (ref. 9) that show redispersion in oxygen and aggregation in reducing conditions, which was further extended e.g. by G. Ferre et al. recently (Catal. Sci Technol., doi: 10.1039/d0cy00732c). Also, on Pt/TiO2 (L. DeRita, et al., Nature Materials volume 18, pages746–751(2019)) in situ studies have been reported. A good additional study is on Rh-based ceria catalysts by Ikemoto et al., also studying the reversible behavior by in situ spectroscopies (PCCP, 2019, doi.org/10.1039/C9CP04625A).

Turning to silver-based catalysts as outlined in this study, also in this case the dynamics has been reported before, also by in situ TEM (e.g., Y. Gao, Catal. Sci. Technol., 10.1039/c7cy00831g, 2017, soot oxidation e.g. D. Gardini et al., Appl. Catal. B, 10.1016/j.apcatb.2015.10.029, 2016). Hence, the novelty of the manuscript needs to be sharpened and it is therefore not acceptable in its present form.

Response: We thank the referee for pointing out the inaccurate expression of "lacking in situ characterizations" used in the paper. We were actually aware of a number of in situ studies on the topic of dispersion/redispersion of catalysts [Refs. 21-28]. Up to now, most of the studies based on in situ microscopy or/and in situ spectroscopy demonstrated an oxidation-induced dispersion/redispersion mechanism. For example, Yasutaka Nagai et al. investigated the oxidative dispersion of Pt-CZY catalyst using combined in situ TEM and XAS, proposing the dispersion process dominated by the formation of highly dispersed PtO_x which strongly interacted with the support [Ref. 22]. However, it was unable to fully understand the correlation between the metal dispersion/redispersion and the corresponding oxidation state during the dynamic process of dispersion/redispersion. As we present in this work, the oxygen adsorption

(lower oxidation degree), likes a ligand in organic chemistry, is able to protect the Ag clusters from sintering. It opens a new way to utilize the common gas (O_2) as the protector. To our best knowledge, our study is the very first work to perform in situ microscopy and spectroscopy under exactly the same conditions which leads us to a solid evidence for the identification of metallic state (to be exactly, nearly metallic state with low surface oxidation degree) during Ag dispersion in O_2.

To be accurate on this point, we have removed all the phrases: "lacking in situ characterizations" in the revised manuscript. Furthermore, we have revised the abstract in the revised manuscript on **Page 2** as "……However, the interplay of gas-metal-support interaction in the dispersion processes, especially the gas-metal interaction has not been well illustrated. …… we unravel a new adsorption-induced dispersion mechanism in such a typical oxidative dispersion process. The strong gas-metal interaction achieved by chemisorption of O_2 on metallic Ag clusters is the internal driving force for dispersion. ……"

We have also revised the main in the revised manuscript on **Pages 3-4** as "……The common consensus on the oxidative dispersion includes the formation of mobile metal oxide species from large metal particles and capture of these species on the support, which is regarded as the most crucial step in the so-called oxidation-induced dispersion mechanism revealed by in-situ TEM and in-situ spectroscopy including near ambient pressure X-ray photoelectron spectroscopy (NAP-XPS) and in-situ X-ray absorption spectroscopy (XAS). Nagai et al. ascribed the redispersion of Pt nanoparticles to the formation of Pt oxide species observed in O_2 at 873 K by in-situ XANES which is due to the strong PtO-support interaction. The underlying mechanism needs to be fully depicted.……" We have cited these papers as the referee suggested above when discussing the dispersion mechanism (see Refs. 21-28).

2) Also other groups like the Schlögl group have been very active in the area of silver catalysed oxidation and need attention. Maybe I overlooked a corresponding reference

as mostly the first authour is only given.

Response: We thank the referee for pointing this out. Schlögl's and Ertl's groups have made great contributions in the fundamental studies of silver-based catalysis including both model- and real-catalysts, and we have cited a few of their papers as refs. 45 and 46 in our manuscript.

To further address their contribution in this field, we have cited one more relevant paper by Schlögl's and Ertl's group in the discussion of oxygen adsorption and silver-catalyzed oxidation reactions. See Refs. 46, 47, 53, 54 in the revised manuscript.

3) The dispersion of Ag and its dispersion is described to occur on reduced Ag species with "adsorbed" oxygen rather than oxidized Ag. However, this claim is not clear for me and also not sufficiently convincing for me, as no quantification of the oxidation state of the Ag-species are performed with XPS (only the position of the peak maximum is presented).

Response: For the quantification of Ag oxidation state, we actually use Auger parameters i.e. the sums of Auger kinetic energy (KE) and photoelectron BE, because XPS core-level of Ag is unable to distinguish its oxide or metallic states. As shown in Figure R5 (adopted from Handbook of X-ray Photoelectron Spectroscopy), the Auger parameters of metallic Ag is around 726 eV, whereas the value for silver oxide is usually lower than 725 eV. The values of the Auger parameters can represent the degree of oxidation in Ag quantitatively, which has been widely adopted in literatures [Ref. 11, 12 in SI].

To make clear this point, we have revised the manuscript on **Page 6**: "(see the description of Auger parameter in the supplementary discussion)", and added description to supplementary discussion in the revised SI on **Page 5**, as "For the quantification of Ag oxidation state, we employed Auger parameters i.e. the sums of Auger kinetic energy (KE) and photoelectron BE, because XPS core-level of Ag is unable to distinguish its oxide or metallic states. As shown in supplementary Fig. 17 (adopted from Handbook of X-ray Photoelectron Spectroscopy),[10] the Auger

parameters of metallic Ag is around 726 eV, whereas the value for silver oxide is usually lower than 725 eV. The values of the Auger parameters can represent the degree of oxidation in Ag quantitatively which has been widely adopted in many other literatures.[11, 12]"

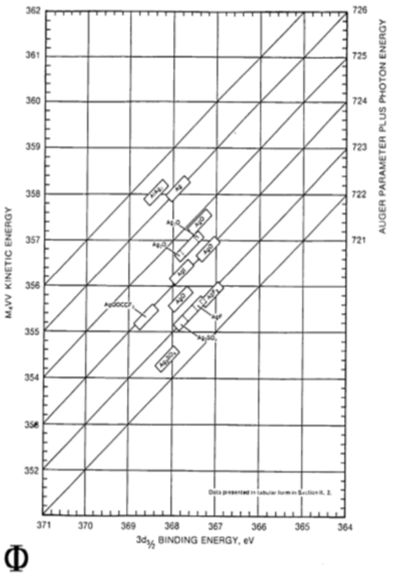

Figure R5. Wagner plots for Ag.

Note also that in the abstract the authors write "…we unravel that in such a typical oxidative dispersion process Ag actually remains in its metallic state and the dispersion is caused by the chemisorption of O_2." A chemisorption – so the formation of a chemical bond - of O_2 is an oxidation if considering cluster/particles. The authors should overthink their wording. They state that Ag is not oxidized, but it is still called a "oxidative dispersion". This is contradictory.

Response: We agree with the reviewer that the chemisorption of O_2 would lead to some charge transfer between Ag clusters and O_2 molecules. However, the difference in the degree of oxidation makes our findings different from the conventional understanding. In the conventional understanding, the dispersed metal atoms/clusters normally have a high oxidation degree (formation of bulk oxides) because they form strong chemical bonds not only to O from the atmosphere but also to the lattice oxygen of the oxide support surface. The later one is considered as a key point to

stabilize the dispersed atoms/clusters [Refs. 12, 21, and 22]. Our work shows that besides the strong metal-support interaction, the dispersion could occur with the assistance of O_2 in the atmosphere. This is also shown in our theoretical model that the dispersion is energetically favored when only 1 out of 3 and 4 out of 8 Ag atoms are bonded to O. Particularly, in the case of Ag_3 cluster the dispersion was caused by the non-dissociative adsorption of molecular oxygen rather than dissociative adsorption of atomic oxygen, which verifies a preferred metallic state under in situ conditions (1 mbar, 673 K).

To make our point clearer, we used the wording of "high" and "low" oxidation degree in the revised manuscript following the reviewer's suggestion. Also see the changes in revised manuscript, "bulk oxidation with a high oxidation degree" on **Page 9**, "nearly metallic clusters with a low surface oxidation degree" on **Page 9**, etc.

4) When reading the manuscript, it becomes not clear that 3 different type of samples were used for the characterization and not only 1 sample. Especially for deducting mechanistic insights, this is to my opinion misleading, since due to the different surface concentrations, the samples can behave as well differently. In the experimental part it is written: "prepared three kind of samples with different Ag density (high, medium, and low). To acquire strong Auger spectra, samples with high NW density were used and shown in Fig. 4. Samples with medium AgNW density were used to get obvious change of Ag/Si ratio during various treatments, also to make XPS peak intensity strong enough, shown in Figs. 1-3."

Response: We thank the referee for pointing this out. Here, the purpose of using three types of samples with different Ag density is only to improve the signal-to-noise ratio of XPS data for presentation, as the intensity drops dramatically under different environmental pressures. In our experiments, the three samples show the same trend of behavior/dynamics during dispersion, despite only slight difference in the dispersion rate (duration to reach equilibrium) and dispersion degree, as verified by XPS in Fig. 1e, Fig. 3b, and Fig. 4c.

To further eliminate the effect of Ag density, we have performed additional ESEM

experiments on the dispersion of Ag nanowires with medium and high density. As shown in Figure R5, both samples are dispersed into small clusters/particles after the O_2 treatment and become invisible under the SEM imaging condition. We can thus safely conclude that the surface Ag density has minor effect on the dispersion dynamics under our experimental conditions.

To address this point, we have put an additional supplementary Fig. 16 (Fig. R6) and supplementary discussion in the SI on **Pages 6 and 15**:

"**Effect of Ag density.** The effect of surface Ag nanowire density can't be ignored when we discuss the dispersion process. As shown by XPS (Fig. 1e, Fig. 3b and Fig. 4c) and SEM (Fig. 1c and supplementary Fig. 14) results, the three samples showed the same trend of behavior/dynamics during dispersion, despite only slight difference in the dispersion rate (duration to reach equilibrium) and dispersion degree. All samples were dispersed into small clusters/particles after the O_2 treatment and become invisible under the SEM imaging condition. We can thus safely conclude that the surface Ag density has minor effect on the dispersion dynamics under our experimental conditions."

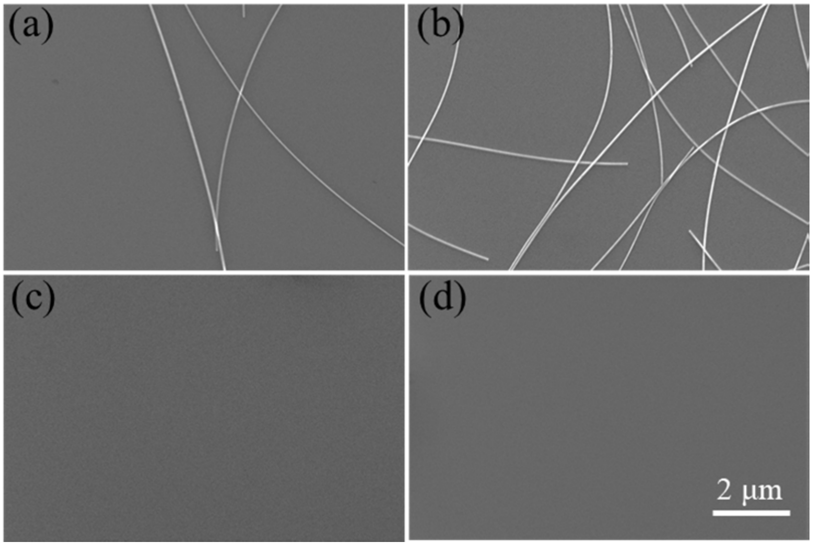

Figure R6. Effect of surface Ag density on the Ag dispersion. (a, c) SEM images of medium density Ag/Si_3N_4 and Ag/Si_3N_4-O-673. (b, d) SEM images of high density Ag/Si_3N_4 and Ag/Si_3N_4-O-673. Scale bar in the insets is 2 μm.

5) Comment to the conclusion: "We provide a solid evidence for the fact that oxide formation is not necessary in oxidative dispersion, but a result during cooling in O_2." This is contradictory to the DFT results, where it was stated that "On the contrary, associative O_2 and dissociative O atoms adsorb stably at the Ag_3-Si_3N_4 ($E_{ads}^{clu} = -2.24$ eV) and Ag_8-Si_3N_4 ($E_{ads}^{clu} = -3.97$ eV) interfaces". The provided data does suggest, that a surface oxidation (nothing else is a O_2 chemisorption of "dissociative O atoms") is needed for the dispersion and not a bulk oxidation. As mentioned before, the wording has to be improved and the terminology should be defined to enhance the clearness of the manuscript.

Response: We thank for the referee's comment. Following the referee's suggestion, here we distinguish a surface oxidation with a low oxidation degree from a bulk oxidation with a high oxidation degree. We have changed the text of "oxide formation" to be "a bulk oxide with a high oxidation degree". See the changes in the revised manuscript as "bulk oxide with a high oxidation degree" on **Page 9**, "AgO_x cluster with high oxidation degree" on **Page 10**, etc.

In summary, the manuscript is not recommended for publication in Nature Communications in its present version because the novelty is not clear enough yet, the process of redispersion (chemisorption/oxidation) and also some experimental details/choice of the experimental study (systematics) are not clear.

Response: Regarding the novelty of our paper, we have demonstrated for the first time two independent processes during dynamic dispersion of Ag: i) metal redispersion is achieved at elevated temperature driven by O_2 adsorption on metallic Ag (surface oxidation of Ag with a low oxidation state) and ii) bulk oxidation of Ag (a high oxidation state) is only as a result of cooling in O_2, which overturns that conventional understanding of oxidation-driven dispersion. It fills in the blanks in the picture of metal dispersion: the interaction between gas molecules and metal surface is as important as the metal-support interaction in some cases. Our findings reveal that the highly dispersed Ag clusters/atoms with a low oxidation state can be stabilized by

O_2 atmosphere during dispersion or even under reaction conditions at 673 K up to 35 h (see time on stream stability test in Fig. R4), and further open up a new route for the fabrication of highly/atomically dispersed catalyst by gas environment during reactions.

Therefore, we believe our results can benefit not only the fundamental understanding of metal dispersion during O_2 and even reaction conditions, but also provide a new route for the fabrication of highly/atomically dispersed catalyst under dynamic reaction conditions.

To further address the novelty, we have revised the manuscripts accordingly on **Page 13** as "The time-on-stream stability test in Fig. 5c further reveals an excellent durability of highly dispersed Ag/Si_3N_4 catalyst under the reaction condition (at 673 K) up to 35 hr (also see supplementary discussion). Our results thus prove the long-term stability of dispersed metallic Ag atoms/clusters under O_2-rich reaction environments, and open up a new route for the fabrication of atomically dispersed catalyst dynamically during reactions." and **Page 15** as "It also opens up a new route to stabilize metal atoms/clusters under realistic reaction conditions, and benefit the fabrication of highly/atomically dispersed catalyst during reactions."

Reviewer #3:

It is an interesting and solid piece of study where in situ XPS technique has been applied to study the "oxidative dispersing" of Ag particles. It was observed that metallic Ag, instead of Ag oxide, was the main species being dispersed. The authors further confirmed that the chemisorption of O_2 is the driving force for the re-dispersion of metallic Ag clusters. While the study is thorough, and self-consistent, its wide applicability and general validity remains a question. What is reported here may represent a special case rather than a general phenomenon.

Response: We thank the referee for the positive evaluation of our paper as a thorough and self-consistent study. To further address the general validity and applicability, we have performed new experiments of Ag dispersion on a wide range of O_2 pressures

and a variety of oxides including widely used SiO_2, Al_2O_3 and $SrTiO_3$ supports. Our point-by-point responses are listed below.

1) First of all, Si_3N_4 is not a typical support. Ag/Si_3N_4 is not a widely used commercial catalyst. Ag/Si_3N_4 is not a representative system. There is no guarantee that the dispersion behaviour of metal on non-oxides to be the same as on oxides.

Response: We agree with the referee that Si_3N_4 is not often used as catalyst support. The choice of this substrate in the work is to make use of the inert property of the Si_3N_4 surface and thus to emphasize the critical role of O_2 atmosphere in the dispersion process. According to the reviewer's suggestion, we have performed new experiments of Ag dispersion on a variety of non-reducible oxides including widely used silica (SiO_2/Si(111)) (Fig. R7a) and sapphire (Al_2O_3(0001)) (Fig. R7b) substrates and also reducible oxide: $SrTiO_3$(100) (Fig. R7c). The same dispersion behavior of Ag (upshift of Ag 3d BE induced by size effect and increasing Ag 3d peak intensity) was clearly observed under oxygen-rich environment as shown in Figure R7, verifying that our findings on Si_3N_4 surfaces is a general phenomenon which can be applied to other oxides. The nearly-metallic state of Ag during dynamic dispersion process was also observed on silica substrate (SiO_2/Si(111)) (Fig. R8).

To address this point, we have added an extra supplementary Fig. 3, 7, and supplementary Table. 1 (Fig. R7 and R8, Table. R1) in the revised SI on **Pages 8 and 10** and revised the manuscript on **Pages 6 and 10** as "To further validate the universality of this dynamic dispersion of Ag on different support materials, we have performed additional experiments on a variety of oxides including widely used SiO_2/Si(100), Al_2O_3(0001) and $SrTiO_3$(110) substrates. The upshift of Ag 3d BE and the increase in the peak intensity were observed on all the substrates (supplementary Fig. 3), similar to that on the Si_3N_4 surface."

"Similar metallic state of dispersed Ag species was also observed on SiO_2/Si(100) in O_2 at elevated temperatures, verifying a universal phenomenon validated for SiO_2 and other oxides (see Supplementary Fig. 7)."

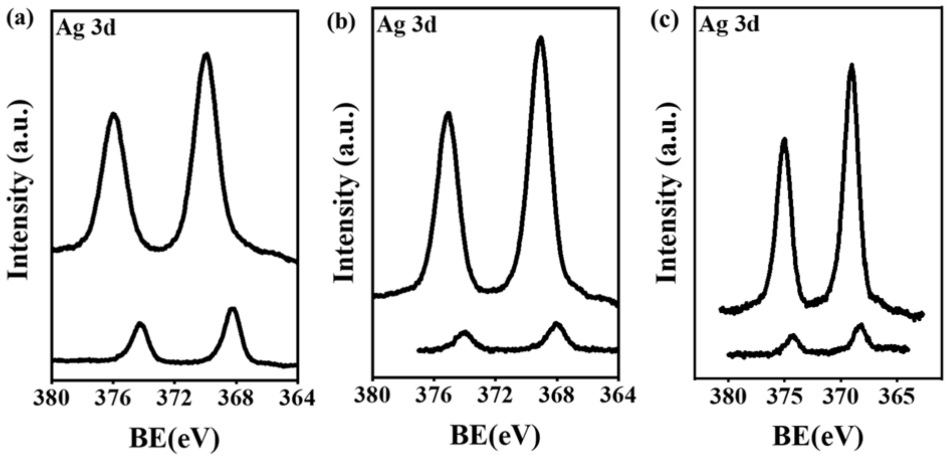

Fig. R7. Dispersion behavior of Ag on different supports. XPS Ag 3d spectra of Ag/oxide surfaces before and after the oxygen treatment in 1 mbar O_2 at 673 K: (**a**) SiO_2/Si(100); (**b**) Al_2O_3(0001) and (**c**) $SrTiO_3$(110). The bottom spectra and upper spectra are the Ag 3d spectra of the Ag/oxide surfaces before and after the oxygen treatment, respectively.

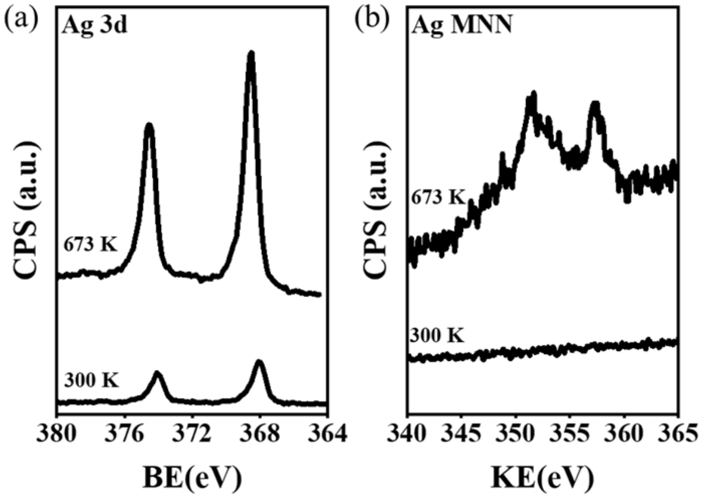

Fig. R8. In-situ XPS study of dispersion behavior of Ag on SiO_2 support. (a) Ag 3d XPS spectra and (b) Ag MNN Auger spectra of Ag-SiO_2 upon annealing in 1 mbar O_2.

Table. R1. XPS results of Ag/SiO_2 surfaces upon annealing in 1 mbar O_2.

Temperature	Ag $3d_{5/2}$/eV	Ag $M_4N_5N_5$/eV	Auger parameter/eV
300 K	368	---	726 (previous results)
673 K	368.5	357.2	725.7

2) Further, oxidative re-dispersion is normally conducted at 1 bar in air. The concentration of O_2 is orders of magnitude higher than the O_2 used in in situ XPS. This will change hugely the oxidation behaviour of Ag. Unless the same observation is obtained on a more typical metal oxide support under conditions closer to general practice, the implication of the study is compromised. Consider doing this requires effort beyond a major revision, I would suggest the authors to go for a more specialized journal. It is a very well conducted piece, and I feel it can be published in any surface chemistry journals with essentially no revision.

Response: We thank the referee for the important comment. Firstly, the universality of a more typical metal oxide support has been fully addressed by performing the same investigation over the Ag nanowires on silica, sapphire and $SrTiO_3$ surfaces, as shown in the response to the former question, as well as in the revised manuscript on **Pages 6 and 10** and the revised SI on **Pages 8 and 10**.

Regarding the O_2 pressure effect, our quasi in-situ XPS experimental results for the 1 bar pressure have indicated the same dispersion behavior (see supplementary Fig. 11). Though XPS experiment for 1 bar pressure is still technically limited by the current NAP-XPS technique, the previous works have confirmed that increasing the O_2 pressure from 1 mbar to 1 bar at 673 K will lead to higher coverage of adsorbed oxygen but not the oxidation (*Phys. Rev. Lett.* 2003, 90, 256102) and Ag_2O decomposes at the temperature of 673 K in air (*Phys. Chem. Chem. Phys.* 2001, 3, 3838-3845). Therefore, from the thermodynamic point of view the same mechanism should be valid in 1 bar O_2 as that in 1 mbar O_2.

To address this point, we have added a paragraph to supplementary discussion in the revised SI on **Page 6**, as "Though XPS experiment for 1 bar pressure is still

technically limited by the current NAP-XPS technique, the previous works have confirmed that increasing the O_2 pressure from 1 mbar to 1 bar at 673 K will lead to higher coverage of adsorbed oxygen but not the oxidation[18] and Ag_2O decomposes at the temperatures of 673 K in air.[19] Therefore, from the thermodynamic point of view the same mechanism should be valid in 1 bar O_2 as that in 1 mbar O_2."

Reviewer #1 (Remarks to the Author):

The authors have replied with suitable statements. Now the paper can be accepted from my side.
